# Subgraphs of functional brain networks identify dynamical constraints of cognitive control

**DOI:** 10.1371/journal.pcbi.1006234

**Published:** 2018-07-06

**Authors:** Ankit N. Khambhati, John D. Medaglia, Elisabeth A. Karuza, Sharon L. Thompson-Schill, Danielle S. Bassett

**Affiliations:** 1 Department of Bioengineering, University of Pennsylvania, Philadelphia, Pennsylvania, United States of America; 2 Department of Psychology, Drexel University, Philadelphia, Pennsylvania, United States of America; 3 Department of Neurology, Perelman School of Medicine, University of Pennsylvania, Philadelphia, Pennsylvania, United States of America; 4 Department of Psychology, University of Pennsylvania, Philadelphia, Pennsylvania, United States of America; 5 Department of Electrical & Systems Engineering, University of Pennsylvania, Philadelphia, Pennsylvania, United States of America; 6 Department of Neurology, University of Pennsylvania, Philadelphia, Pennsylvania, United States of America; 7 Department of Physics & Astronomy, University of Pennsylvania, Philadelphia, Pennsylvania, United States of America; Oxford University, UNITED KINGDOM

## Abstract

Brain anatomy and physiology support the human ability to navigate a complex space of perceptions and actions. To maneuver across an ever-changing landscape of mental states, the brain invokes cognitive control—a set of dynamic processes that engage and disengage different groups of brain regions to modulate attention, switch between tasks, and inhibit prepotent responses. Current theory posits that correlated and anticorrelated brain activity may signify cooperative and competitive interactions between brain areas that subserve adaptive behavior. In this study, we use a quantitative approach to identify distinct topological motifs of functional interactions and examine how their expression relates to cognitive control processes and behavior. In particular, we acquire fMRI BOLD signal in twenty-eight healthy subjects as they perform two cognitive control tasks—a Stroop interference task and a local-global perception switching task using Navon figures—each with low and high cognitive control demand conditions. Based on these data, we construct dynamic functional brain networks and use a parts-based, network decomposition technique called non-negative matrix factorization to identify putative cognitive control subgraphs whose temporal expression captures distributed network structures involved in different phases of cooperative and competitive control processes. Our results demonstrate that temporal expression of the subgraphs fluctuate alongside changes in cognitive demand and are associated with individual differences in task performance. These findings offer insight into how coordinated changes in the cooperative and competitive roles of cognitive systems map trajectories between cognitively demanding brain states.

## Author summary

Brain networks support the human ability to navigate a complex space of perceptions and actions through cognitive control. Here we ask, “How do brain networks coordinate task-relevant information as individuals adapt to cognitive demands imposed by a task?” We study the fMRI BOLD signal of twenty-eight healthy subjects as they perform two cognitive control tasks—a Stroop interference task and a local-global perception switching task using Navon figures—with low and high cognitive load conditions. We construct functional networks and use a machine learning technique called non-negative matrix factorization to identify topological motifs whose expression fluctuates across different phases of cognitive control processes. We find that motifs stratify the brain network into a hierarchy of distributed functional processes that adapt to changes in cognitive demand and predict individual differences in task performance. These data offer insight into how network interactions linking cognitive systems coordinate transitions between cognitively demanding brain states.

## Introduction

**Fig 1:**
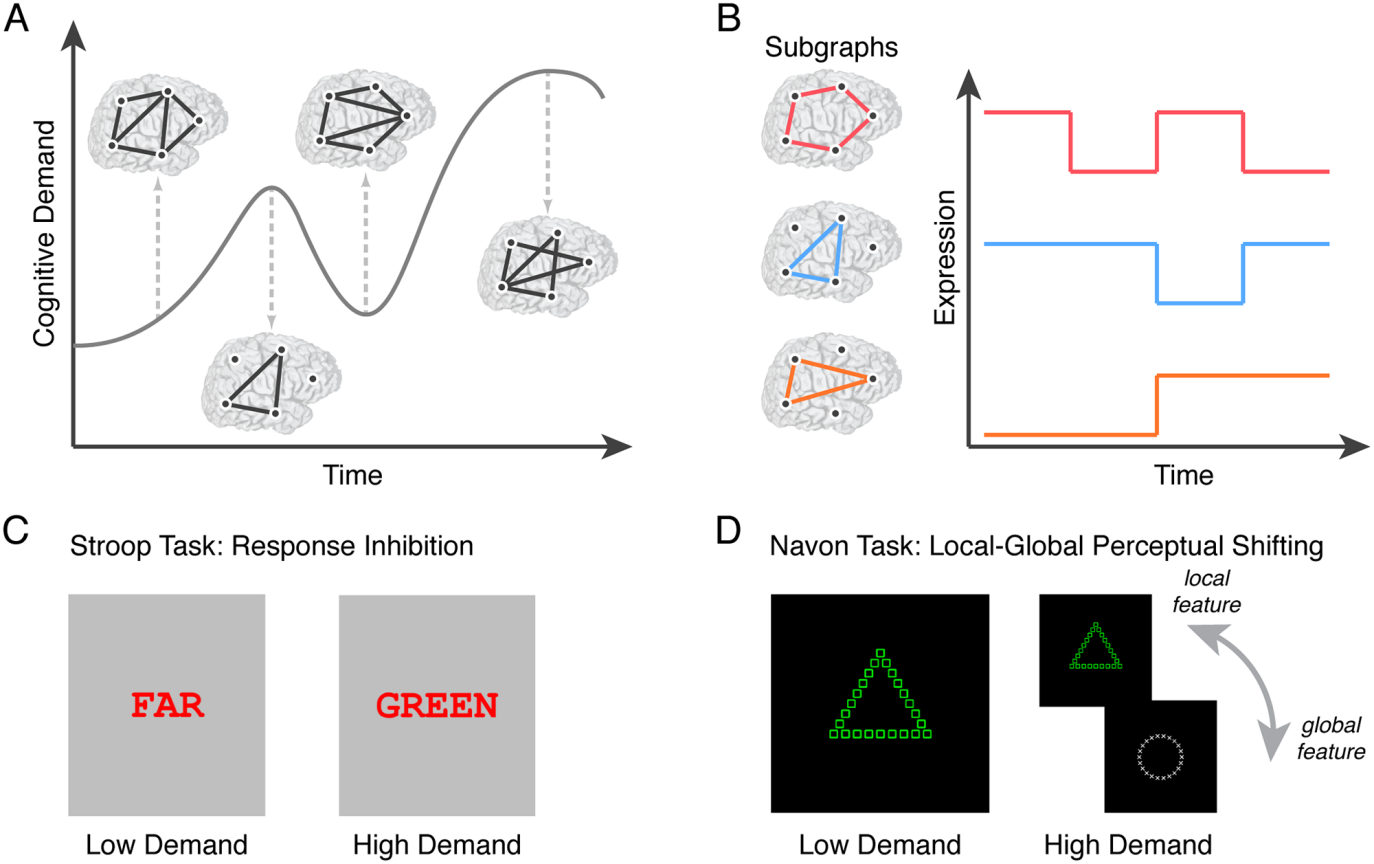
Experimentally modulating cognitive control processes to uncover internal mechanisms of network regulation. (*A*) To monitor and regulate the demands placed on neural systems, empirical evidence suggests that the brain employs *cognitive control* processes that gate information and select among competing representations and processes [14]. Functional brain networks that flexibly coordinate interactions between different sets of brain regions over time may be a key substrate for cognitive control, and moreover be essential for maintaining homeostasis between internally-driven brain dynamics and externally-elicited behavioral goals [8]. We present here a conceptualized diagram of the graph theoretical framework that helps us model the dynamics of cognitive control networks. Brain regions are represented as *nodes* and the strength of functional interactions between brain regions are represented as *weighted edges*. (*B*) Recent advances in network neuroscience [15] and machine learning [16] enable us to cluster functional brain networks into composite subgraphs—cohesive sets of graph edges (*left*) from the observed network (*A*) that tend to co-vary in strength over time. The putative role of a subgraph in cognitive control is inferred by its relative level of weighted expression in the observed network at a specific task block during cognitive processing (*right*). To experimentally modulate cognitive demand, we recruit 28 healthy adult human participants to perform a response inhibition, Stroop task (*C*) and a task-switching, local-global feature perception task based on Navon figures (*D*). The Stroop task entails (i) a fixation condition consisting of a black crosshair at the center of the screen, (ii) a low demand condition consisting of a matched word-color pair, and (iii) a high demand, interference condition consisting of a mismatched word-color pair. Subjects are required to report the color of the presented word. The Navon task entails (i) a fixation condition consisting of a black crosshair at the center of the screen, (ii) a low demand condition consisting of only white or green Navon figures—local shapes embedded in a non-matching global shape, and (iii) a high demand condition consisting of Navon figures randomly alternating between white or green color. Subjects are required to report the local shape if the presented figure is white or to report the global shape if the presented figure is green. Differences in task condition are thought to invoke different levels of recruitment of cognitive control mechanisms. Participant reaction time on correct trials is used to measure performance, and the difference in performance between high and low cognitive control conditions is thought to represent the costs of cognitive control.

In human cognition, internally-generated *cognitive control* processes modulate attention, facilitate task switching, and inhibit prepotent behavior [1]. One avenue by which the brain may rapidly traverse a cognitive state-space is through its functional interactions—coherent fluctuations in brain activity shaped by the structural connectome [2]. The brain’s distributed functional interactions form a functional network whose architecture is temporally dynamic [3], conferring adaptivity in the face of environmental pressures or task demands [4] such as those elicited during learning [5] and other tasks demanding executive cognition [6]. Cognitive control processes have been widely reputed to recruit several cognitive systems that include executive, attention, and salience systems that span prefrontal cortices, striatum, parietal regions, and cerebellum [7–12]. The notion that cognitive control involves a heterogenous collection of brain systems is supported by several univariate studies demonstrating concurrent activation of functionally-specialized brain areas across different cognitive control tasks [13]. If patterns of measured brain activity signal involvement of different brain regions across a diverse set of cognitive control tasks, then how do functional brain networks encode and coordinate this task-relevant information to adapt to fluctuations in cognitive demand (Fig 1A)?

One mechanistic theory, known as the “adaptive coding model of cognitive control” [17], posits that brain regions that activate during higher cognitive functions can alter their dynamical properties based on the current goals of the neural system. More recent studies have challenged this hypothesis by presenting data that suggests that changes in the cognitive demands of a task lead to recruitment of mechanistically-specialized brain regions based on an anatomically-defined gradient [18, 19]. To reconcile these opposing theories of the neuronal basis of cognitive control, [20] applied multivoxel pattern analysis—a machine learning technique for identifying consistent patterns of voxel-wise activation—to the fMRI of subjects as they performed simple and cognitively demanding tasks. The authors found a consistent pattern of activation in frontoparietal brain areas that was specific to highly demanding conditions across multiple cognitive tasks. Their findings support the hypothesis that a consistent group of brain regions activate in response to increases in cognitive demand. However, parallel lines of investigation on the underpinnings of cognitive control in functional brain networks suggest that the integrated cognitive control network dissociates into several, segregated sub-networks that are responsible for different aspects of cognitive control processes [13]. To address these conflicting reports, a data-driven approach that can disentangle parts of functional brain networks that encode cognitive states associated with control tasks—and track their expression alongside changes in cognitive demand—is required. Such a capability would improve our understanding of which components of functional brain networks are important for different facets of cognitive control, and how these components encode shifts between cognitively demanding states.

In the present work, we identify components of functional brain networks associated with transitions between cognitively demanding states by using an unsupervised machine learning technique known as non-negative matrix factorization (NMF) [21]. NMF decomposes functional brain networks into: (i) additive subgraphs that represent clusters of graph edges that track with one another over time, and (ii) time-varying coefficients that quantify the degree to which a subgraph is expressed at a given point in time [16, 22, 23]. This computational tool allows us to track how groups of functionally interacting brain areas are dynamically expressed during experimentally modulated changes in cognitive demand (Fig 1B; a discussion regarding the differences between NMF and components analysis can be found in textitMaterials and methods). In particular, we ask participants to engage in the following two cognitive control tasks: a response inhibition Stroop task (Fig 1C; [24]) and a local-global perception switching task based on classical Navon figures (Fig 1D; [25]). Our methodological approach enables us to address a critical question in cognitive control: “How do brain networks coordinate task-relevant information as individuals adapt to the cognitive demands imposed by a task?”

To address this question using NMF, we draw upon recent studies that suggest that task-driven reconfiguration of functional brain networks integrates otherwise functionally-specialized and segregated information [26, 27]. One compelling current theory proposes that transitions between cognitively demanding brain states are facilitated by dynamic changes in the patterns of correlated and anticorrelated brain activity such that anticorrelated fluctuations in brain activity represent segregated brain functions, and correlated fluctuations in brain activity represent integrated brain functions [28, 29]. Correlated and anticorrelated dynamics may explain how task-relevant information is shared between different regions of the network during cognitively demanding tasks. In this study we construct functional brain networks by applying the Pearson correlation function to block level fMRI collected during cognitive control tasks. By accounting for correlated and anticorrelated functional interactions in the NMF framework, we can determine the likelihood that the functional interactions within a subgraph are collectively correlated or anticorrelated at a particular point in time—providing a perspective on integrated and segregated information processing among composite sets of brain regions.

Based on prior studies demonstrating that behavioral tasks can be used to dissociate intrinsic and task-specific architectures of functional brain networks [30], we first hypothesize that NMF will identify functional subgraphs whose expression is either generalized across the Stroop and Navon tasks or specific to distinct cognitive conditions within and between tasks. In particular, we expect task-general subgraphs to reflect interactions relevant for task saliency and cognitive control processes common to both tasks. We also expect task-specific subgraphs to reflect interactions relevant for stimulus processing and attentional mechanisms necessary for either response inhibition in the Stroop task or task-switching in the Navon task. Building upon recent evidence that functional interactions dynamically reorganize between integrated and segregated network states [26], we next hypothesize that functional subgraphs will shift their roles between correlated and anticorrelated modes of interaction in response to experimentally driven changes in cognitive demand. Lastly, we hypothesize that changes in subgraph expression during experimental modulation of cognitive demand will reflect inter-individual differences in behavioral performance on the task. Specifically, based on previous theories regarding the behavioral influence of correlated and anticorrelated functional interactions in cognitive control [29], we expect that components of the frontoparietal and default mode systems will most prominently participate in subgraphs associated with individual differences in performance.

## Results

### Decomposing functional subgraphs of cognitive control

**Fig 2:**
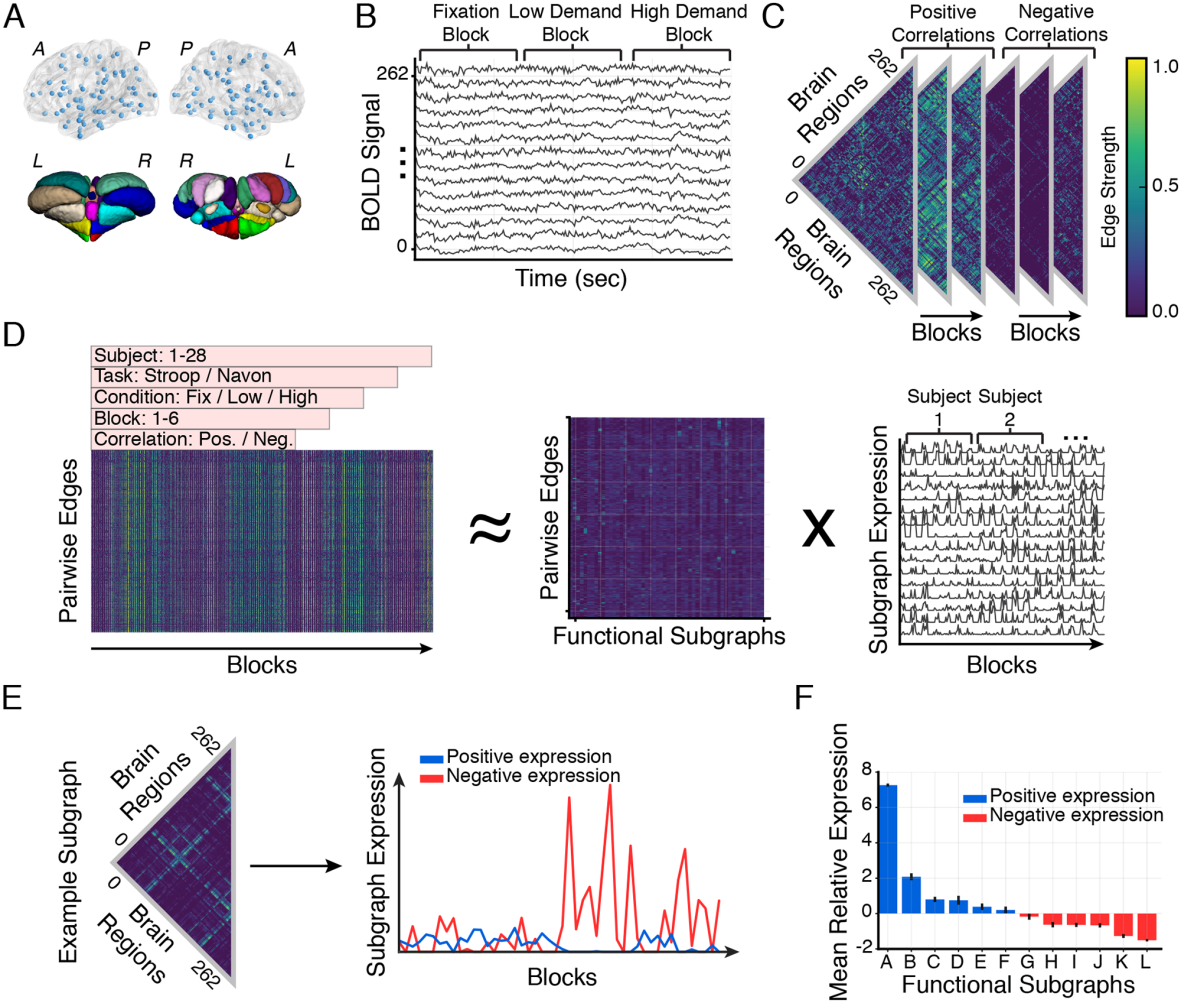
Learning functional subgraph architecture of cognitive control processes. (*A*) We measure fMRI BOLD signals from 262 functional regions of interest (234 cortical and subcortical brain areas parcellated by ([31]; *top*) and 28 cerebellar brain areas parcellated by ([32]; *bottom*) as 28 healthy adult human subjects perform Stroop and Navon cognitive control tasks. (*B*) We concatenate BOLD signal from 6 task blocks (corresponding to 30 seconds of BOLD activity, and comprising several trials) in each of 3 task conditions (fixation, low demand, high demand) for each of 2 tasks (Stroop and Navon). (*C*) Next, we calculate the Pearson correlation coefficient between each pair of regional BOLD signals to create an adjacency matrix for every experimental block. We encode this information in dynamic functional networks with brain regions as graph nodes and block-varying correlation as weighted graph edges. To assess the relative role of correlated (positively weighted edges) and anticorrelated (negatively weighted edges) functional interactions during cognitive control, we threshold each adjacency matrix at the zero edge weight and group positive edges and negative edges into separate adjacency matrices (see Materials and methods). (*D*) We concatenate all pairwise edges over task blocks and subjects, and we generate a single network configuration matrix for the entire study cohort (*left*). We apply non-negative matrix factorization (NMF)—a parts-based decomposition of the dynamic network—to the configuration matrix and cluster graph edges with co-varying weights into a matrix of subgraphs (*middle*) and a matrix of time-varying coefficients (*right*) that quantify the level of expression of each subgraph in each task block. We use a cross-validation parameter optimization procedure and identify 12 subgraphs specific to the cognitive control tasks (S2 Fig). (*E*) For each subgraph, we reconstitute its vector of edge weights into a fully-weighted symmetric adjacency matrix (*left*) and track its associated positive and negative expression coefficients over task blocks (*right*). Briefly, the positive and negative expression coefficients signify the likelihood that the subgraph edges represent correlations or anticorrelations for each moment in time (see Materials and methods). (*F*) We rank functional subgraphs in decreasing order (A-L) of the difference between positive and negative expression weight, averaged over task blocks and subjects. Bar height represents the mean difference over subjects and error bars represent standard error of the mean. Red bars correspond to subgraphs that are, on average, more positively expressed and blue bars correspond to subgraphs that are, on average, more negatively expressed.

To uncover the topological organization and putative roles of correlated and anticorrelated functional interactions in cognitive control, we first acquire fMRI data as 30 healthy adult human subjects perform Stroop and Navon cognitive control tasks. Two subjects are excluded on the basis of poor performance and technical problems on the day of scanning, leaving 28 subjects for further analysis. In particular, we measure fMRI BOLD signals from 262 functional brain areas (Fig 2A)—including cortex, subcortex, and cerebellum [31, 32]—during three separate conditions of both the Stroop and Navon tasks: fixation, low cognitive demand, and high cognitive demand conditions (Fig 2B). Briefly, the low cognitive demand condition is designed to elicit a neural response associated with performing each task with low cognitive control demands and the high cognitive demand condition is designed to elicit a neural response associated with either task shifting or inhibition cost (see Material and methods for more details). We then construct dynamic functional brain networks for each subject where network nodes represent brain regions and network edges represent the Pearson correlation coefficient between regional BOLD time series (Fig 2C). Specifically, we compute a 262 × 262 adjacency matrix for each of 6 task blocks (corresponding to 30 seconds of BOLD activity, and comprising several trials) in each of the 3 task conditions (fixation, low demand, high demand) for each of 2 tasks (Stroop and Navon). This process results in 36 block-level adjacency matrices per subject. Importantly, positive Pearson correlations underlie integrated and coherent activation between brain regions or *correlated functional interactions*, and negative Pearson correlations underlie segregated and discordant activation between brain regions or *anticorrelated functional interactions* [28]. To separate positively-weighted network edges (correlated interactions) from negatively-weighted network edges (anticorrelated interactions), we duplicate the adjacency matrix of each block and separately threshold edge weights either greater than zero or less than zero (see Materials and methods for details). Lastly, we aggregate all functional brain networks into a network configuration matrix (Fig 2D) with size 2016 × 34191. The first dimension of size 2016 corresponds to all combinations of two tasks, three task conditions, six repeated blocks, twenty-eight subjects, and two edge types (correlated or anticorrelated); the second dimension of size 34191 corresponds to all unique, pairwise edges between the 262 brain regions.

We first assess the extent to which task-specific differences in functional network topology are explained by first-order, global network statistics, by comparing the distribution of mean edge strengths across different dimensions of the network configuration matrix (S1 Fig). We find no significant difference in mean edge strength across subjects between blocks during the Stroop task and blocks during the Navon task (paired *t*-test, *t*_27_ = −1.5, *p* = 0.14; S1B Fig). We also find no significant difference in mean edge strength across subjects between blocks during the low cognitive demand condition and blocks during the high cognitive demand condition (paired *t*-test, *t*_27_ = 0.35, *p* = 0.73; S1D Fig). We find a significant decrease in mean edge strength between blocks during the fixation period and blocks during the cognitive control task period (paired *t*-test, *t*_27_ = 4.7, *p*6.3 × 10^−5^; S1C Fig), suggesting that engaging in cognitive control tasks is associated with a reduction of correlated and anticorrelated BOLD dynamics. Critically, this result suggests that attention-related brain states thought to be associated with the fixation period may be subserved by stronger and more well-defined functional relationships between brain regions than more complex, task-driven brain states. Overall, our findings suggest that differences in functional network topology during cognitive control tasks and control conditions are not driven by first-order differences in mean edge strength of the network. Rather, we expect that differences in the topological organization of correlated and anticorrelated BOLD dynamics may be complex and heterogenously distributed across the functional network.

To disentangle patterns of correlated and anticorrelated BOLD dynamics related to cognitive control processes, we extract functional subgraphs and their dynamic expression from functional brain networks. Specifically, we apply an unsupervised machine learning algorithm called non-negative matrix factorization (NMF) to the network configuration matrix. This technique enables us to pursue a parts-based decomposition of network edges into additive functional subgraphs with accompanying expression coefficients that measure the degree to which the subgraph is expressed in a particular task block, task condition, subject, and edge type (Fig 2D) [22, 23]. Each subgraph composes a 262 × 262 adjacency matrix and each subgraph’s expression coefficients compose a vector of length 2016. Thus, subgraphs detail topological components of the functional brain network and temporal coefficients quantify their expression during different phases of the cognitive control tasks. Moreover, each subgraph is associated with a positive expression component associated with correlated BOLD dynamics and a negative expression component associated with anticorrelated BOLD dynamics (Fig 2E). A critical step in using NMF is optimizing model parameters (number of subgraphs *m*, sparsity of subgraph edge weights *β*, and regularization of temporal expression coefficients *α*) to ensure generalizability of component subgraphs without overfitting the model on observed data. By designing a four-fold, leave-seven-subjects-out cross-validation scheme, we minimize the average cross-validation error on held-out subjects and find the optimal number of subgraphs to be twelve, the subgraph sparsity to be 0.29, and the regularization of the temporal expression coefficients to be 0.56 (S2 Fig; see Materials and methods for more details). For a quality check on the effect of motion confounds on subgraph expression, we refer the reader to S3 Fig. For a test-retest reliability assessment of subgraph reproducibility we refer the reader to S4 Fig.

We next rank the twelve subgraphs (*A-L*) in decreasing order of their relative positive or negative expression across all conditions in the cognitive control tasks. Specifically, we compute the difference between the positive expression coefficient corresponding to correlated dynamics and the negative expression coefficient corresponding to anticorrelated dynamics for each task block and average the difference across blocks of each subject (Fig 2F). Intuitively, subgraphs whose mean relative expression values are positive are more likely to capture correlated BOLD dynamics and subgraphs whose mean relative expression values are negative are more likely to capture anticorrelated BOLD dynamics. We refer to specific subgraphs according to their assigned letter for the remainder of the study.

**Fig 3:**
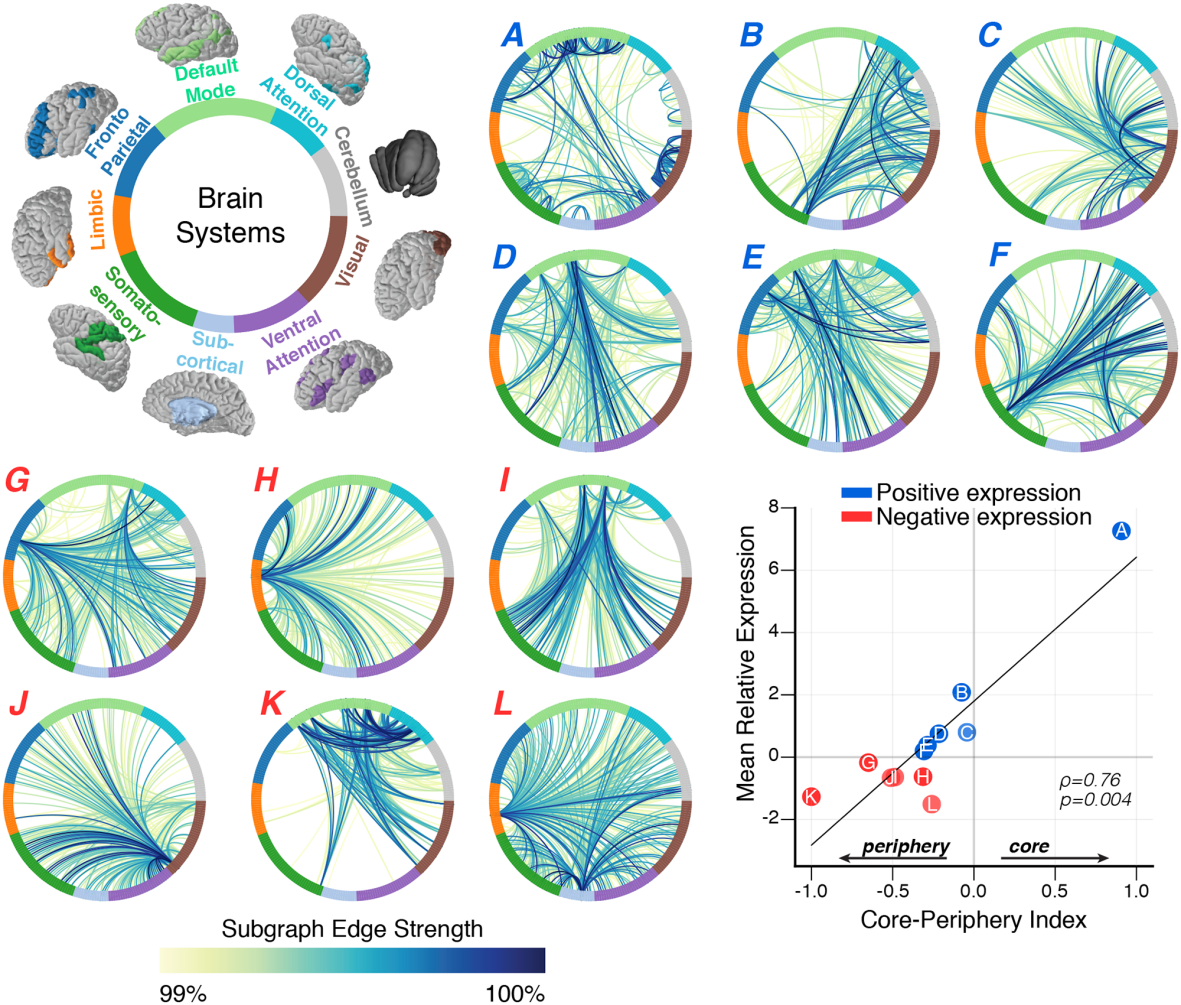
Linking functional subgraphs to neuroanatomy of canonical cognitive systems. We uncover twelve functional subgraphs whose weighted graph edges span the 262 graph nodes specified by the brain atlas. We examine functional roles of subgraphs in cognitive processing by assigning each node to a putative cognitive system [33]: dorsal attention, default mode, frontoparietal, limbic, somatosensory, subcortical, ventral attention, visual, and cerebellum. To visualize the topology of each subgraph, we construct ring graphs in which nodes are evenly spaced around the circumference of a circle—color coded by assigned cognitive system—and edges between nodes are represented by line arcs—colored by the percentile of the edge strength in the subgraph. Subgraphs are coded *A* through *L* in decreasing order of the mean relative expression weight; subgraphs expressed more positively are represented with red letters and subgraphs expressed more negatively are represented with blue letters. For system-by-system adjacency matrix representations of functional subgraphs, we refer the reader to S5 Fig. Subgraphs reflect topological states of task-related processes whereby functional interactions within a cognitive system mark a centralized network *core* of information that may be shared with other cognitive systems located in the network *periphery*. To identify core-periphery structure for a subgraph, we compute the relative difference between the mean weight of edges adjoining nodes within a cognitive system and the mean weight of edges adjoining nodes from that cognitive system to other cognitive systems—values closer to +1 indicate stronger edges within a cognitive system than between cognitive systems (core), values closer to −1 indicate stronger edges between cognitive systems than within a cognitive system (periphery), and values closer to 0 indicate equally strong edges within and between cognitive systems (core-periphery). We observe a significant positive relationship between a subgraph’s core-periphery index and its mean relative expression across task blocks and subjects (Spearman’s *ρ*, *ρ* = 0.76, *p* = 0.004), suggesting that subgraph topology is closely linked with subgraph dynamics. Specifically, subgraphs that exhibit greater core and core-periphery structure express more correlated dynamics (positive; red) and subgraphs that exhibit greater periphery structure express more anticorrelated dynamics (negative; blue). These results imply that subgraphs may represent putative stages of cognitive control in which correlated dynamics correspond to integrated information processes within and across cognitive systems and anticorrelated dynamics correspond to segregated information processes between different cognitive systems. For a detailed comparison of core-periphery structure across cognitive systems and subgraphs, we refer the reader to S6 Fig.

We next ask whether the functional subgraphs expressed during the cognitive control tasks reflect functional interactions within and across known cognitive systems. To study the relationship between the functional subgraph architecture and known cognitive brain systems, we assign each of the 262 brain regions into one of nine cognitive systems [33]: dorsal attention, default mode, frontoparietal, limbic, somatosensory, subcortical, ventral attention, visual, and cerebellum. Thus, we re-organize the rows and columns of each subgraph’s 262 × 262 adjacency matrix such that nodes assigned to the same brain system are contiguously ordered, and we visualize the resulting adjacency matrices as circular, ring graphs (Fig 3; for matrix representation see S5 Fig). To quantitatively confirm that each subgraph captures functional interactions that are indeed distributed within and between cognitive systems, we compare the average subgraph edge weight between pairs of nodes of the same or different cognitive systems to a null distribution of the average subgraph edge weight—constructed by permuting subgraph edge weights between nodes and recomputing the average subgraph edge weight for each pair of cognitive systems for 10000 permutations. We find that functional subgraphs cluster interactions between brain regions of the same cognitive system and between brain regions of different cognitive systems (*p* < 0.05; Bonferroni corrected for multiple comparisons; see S5 Fig)—implicating a distributed functional architecture underlying the cognitive control tasks. In other words, the functional subgraphs recovered by NMF span several cognitive brain systems defined *a priori* [33].

Based on the distribution of subgraph edges within and between known cognitive systems, we examine how subgraph topology might underlie different information processes during cognitive control. Interconnected complex systems that underlie distributed information processes—such as those involved in cognitive control—can exhibit *core-periphery* structure in which a strongly interconnected *core* of nodes is connected to other nodes in the network *periphery*, which tend to solely connect with core nodes and remain otherwise isolated from other network regions [34, 35]. Intuitively, the putative function of the network core is to integrate information from different, specialized systems located in the network periphery [36, 37]. The core-periphery model has also been extended to accommodate dynamic functional networks in which the network core exhibits less flexible functional connectivity and the network periphery exhibits more flexible functional connectivity [36]. A critical assumption of recent applications of the core-periphery model is that there is a single set of core regions and a single set of periphery regions. It is plausible that brain networks consist of multiple core structures [37–39] that are activated based on ongoing cognitive processes reflected by network subgraphs. To identify core-periphery organization in a subgraph, we compute a core-periphery index (see Methods) that quantifies the difference between mean edge strength within a cognitive system (network core) and mean edge strength between a cognitive system and all other systems, averaged for each cognitive system. Intuitively, the core-periphery index ranges between −1—stronger edges in the periphery than in the core—and + 1—stronger edges in the core than in the periphery; index values closer to 0 imply equally strong edges in the core and in the periphery characteristic of traditional core-periphery structure. We use a surrogate subgraph model (10000 rewiring permutations) to statistically test whether each cognitive system of each subgraph exhibits core and periphery architecture (details regarding specific cognitive systems significantly involved in each subgraph may be found in S6 Fig. We find that different functional subgraphs exhibit varying degrees of core-periphery organization (Fig 3). For example, subgraph A expresses significant core connectivity in seven of nine cognitive systems and significant periphery connectivity in one of nine cognitive systems (S6 Fig). In contrast, subgraph K expresses significant core connectivity in zero of nine cognitive systems and significant periphery connectivity in three of nine cognitive systems (S6 Fig). Intuitively, subgraph A reflects core organization where systems exhibit more centralized topology and subgraph K reflects periphery organization where systems exhibit more decentralized topology. We find that cognitive systems in the remaining subgraphs tend to exhibit both internally centralized connectivity as well as decentralized connectivity to other systems. The differentiation of subgraphs into constituent core-periphery architectures suggests that subgraphs may reflect different modes of integrated and segregated network processes in which task-relevant information may be organized within core cognitive systems and shared with cognitive systems in the network periphery.

Logically, we next ask the question “How does the core-periphery organization of a functional subgraph relate to its dynamical expression during cognitive control?” To answer this question, we examine the relationship between the core-periphery index of a subgraph and its mean relative expression. We hypothesize that functional subgraphs with stronger edges adjoining brain regions in the network core (core-periphery index closer to +1) are expressed more positively and functional subgraphs with stronger edges adjoining brain regions between the network core and network periphery (core-periphery index closer to −1) are expressed more negatively. Intuitively, subgraphs with stronger edges within the core and weaker edges between the core and periphery will be associated with more correlated BOLD dynamics underlying states of integrated cognitive processes, and subgraphs with stronger edges between the core and periphery and weaker edges within the core will be associated with more anticorrelated BOLD dynamics underlying states of segregated cognitive processes. Using the Spearman’s *ρ*, we find a significant positive correlation between core-periphery index and relative subgraph expression (*ρ* = 0.76, *p* = 0.004; Fig 3). This result supports the hypothesis that subgraphs with greater sensitivity to topology within the network core tend be positively expressed and subgraphs with greater sensitivity to topology between the network core and network periphery tend to be negatively expressed. Importantly, we observe that functional subgraphs with more evenly balanced core-periphery topology (core-periphery index close to 0) also tend to be more positively expressed. Collectively, our findings demonstrate that subgraphs with strong core topology or balanced core-periphery topology are associated with network states in which brain regions exhibit correlated dynamics and that subgraphs with strong periphery topology are associated with network states in which brain regions exhibit anticorrelated dynamics. By examining the relationship between subgraph topology and subgraph expression, we may now begin to bridge theoretical interpretations of subgraph architecture with experimentally driven and empirically observed changes in cognitive brain state.

### Recruitment of functional subgraphs during cognitive control tasks

Based on the set of twelve functional subgraphs and their time-varying expression, we next ask “Are functional subgraphs differentially recruited during separate cognitive control tasks?” We hypothesize that a functional subgraph is either sensitive to cognitive control processes specific to each task or to cognitive control processes that are shared between the two tasks. To motivate our hypothesis, we examine relative differences in the distributions of mean strength of each edge between all task blocks of the Stroop task and all task blocks of the Navon task (S7 Fig), before extracting functional subgraphs using NMF. Specifically, we compare the strength of an edge during the Stroop task to its strength during the Navon task by computing the mean difference of Fisher’s *r*-to-*Z* transformed correlations across subjects, separately for positive correlations and negative correlations. We observe stronger positive correlations within and between the dorsal attention, visual, and cerebellar systems during the Navon task than during the Stroop task, and we observe stronger negative correlations between the default mode system and dorsal attention, visual, and cerebellar systems during the Navon task than during the Stroop task.

**Fig 4:**
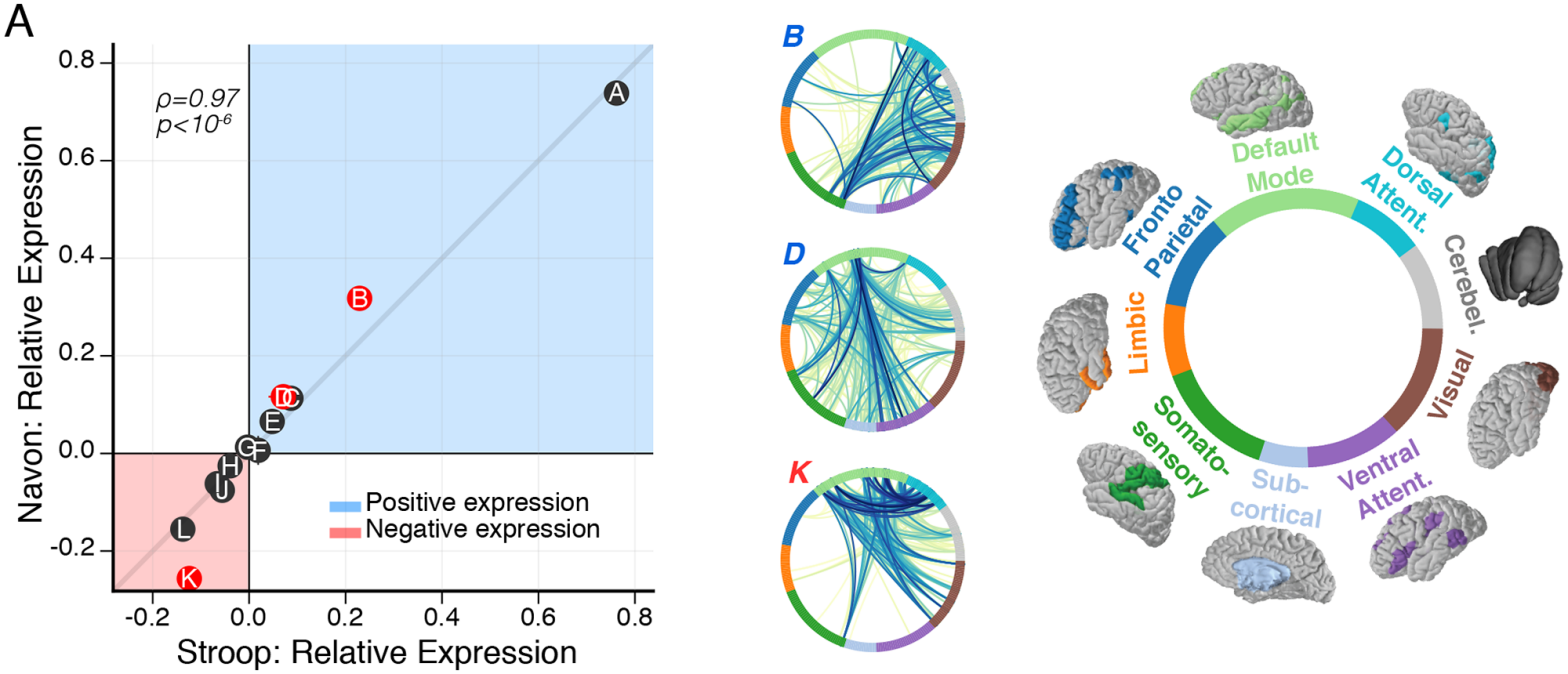
Subgraphs map functional interactions specific to cognitive control tasks. (*A*) Relationship between the relative subgraph expression during the Stroop task and the relative subgraph expression during the Navon task. Each point represents relative subgraph expression averaged over subjects, horizontal (vertical) error bars represent standard error of the mean for the Stroop (Navon) task. Generally, relative subgraph expression during the Stroop task is significantly associated with relative subgraph expression during the Navon task (Spearman’s *ρ*, *ρ* = 0.97, *p* = 1.3^−7^), implying that subgraphs collectively follow similar rules of dynamical expression during the Stroop and Navon tasks. However, individual subgraphs may vary in the amount they are expressed during the Stroop and Navon tasks, which is signified by the perpendicular distance between a subgraph and the shaded gray line with slope equal to one. Using paired *t*-tests and FDR correction for multiple comparisons, we compare the distribution of relative subgraph expression between Stroop and Navon tasks across subjects. We find greater positive expression during the Navon task than the Stroop task for subgraph *B* (*t*_27_ = 4.4, *p* = 1.4 × 10^−4^) and subgraph *D* (*t*_27_ = 2.9, *p* = 7.0 × 10^−3^), and we find greater negative expression during the Navon task than the Stroop task for subgraph *K* (*t*_27_ = 5.1, *p* = 1.4 × 10^−5^). Thus, the rank of a subgraph in terms of its overall expression relative to other subgraphs is similar between the Stroop and Navon tasks, but its level of expression may be different depending on the task. Specifically, we find subgraphs *B* and *D* are more strongly associated with correlated dynamics during the Navon task than the Stroop task, and we find subgraph *K* is more strongly associated with anticorrelated dynamics during the Navon task than the Stroop task.

We use these results to inform our expectation regarding cognitive systems that might be involved in task-specific functional subgraphs. We examine the relationship between the mean relative expression of a subgraph during the Stroop task and the mean relative expression of a subgraph during the Navon task (Fig 4). We find that the expression of a subgraph during the Stroop task is significantly associated with its expression during the Navon task (Spearman’s *ρ*, *ρ* = 0.97, *p* = 1.3 × 10^−7^). This result suggests that subgraphs are similarly ranked based on their respective expression values between the two tasks. Critically, this result implies that subgraph expression may follow a consistent hierarchy of expression during two different cognitive control tasks.

While the relative relationships between subgraph expression are preserved between the Stroop task and the Navon task, we also identify differences in the magnitude of subgraph expression between the tasks. Specifically, we compare the distribution of relative subgraph expression between the Stroop task and the Navon task for each subgraph. Using paired *t*-tests and FDR correction for multiple comparisons, we find greater positive expression during the Navon task than during the Stroop task for subgraph *B* (*t*_27_ = 4.4, *p* = 1.4 × 10^−4^) and subgraph *D* (*t*_27_ = 2.9, *p* = 7.0 × 10^−3^), and we find greater negative expression during the Navon task than during the Stroop task for subgraph *K* (*t*_27_ = 5.1, *p* = 1.4 × 10^−5^). These findings suggest that (i) the Navon task exhibits greater correlated BOLD dynamics within and between dorsal attention, visual, and cerebellar systems (subgraph *B*) and between the default mode system and other broadly distributed cognitive systems (subgraph *D*) than the Stroop task, and (ii) the Navon task exhibits greater anticorrelated BOLD dynamics between the default mode system and dorsal attention, visual, and cerebellar systems (subgraph *K*) than the Stroop task. Critically, subgraph *D* and subgraph *K* both capture functional relationships between the default mode system and other cognitive systems. However, they exhibit different types of interactions—correlated versus anticorrelated—and involve different sub-regions of the default mode system that engage or disengage with other cognitive systems. This heterogeneity may underlie a multi-faceted functional role for cognitive systems involved in both positively expressed and negatively expressed subgraphs as regions of information integration and information segregation during these tasks.

To summarize, our results imply that functional subgraphs follow a general hierarchy of expression during two cognitive tasks that invoke different control processes—pre-potent response inhibition during the Stroop task and perceptual, rule-based task switching during the Navon task. While this hierarchy may establish a task-general functional network organization related to complex cognitive processes, specific processes associated with different forms of cognitive control may be represented through small deviations in subgraph expression that significantly differ between tasks. Accordingly, nine of the twelve subgraphs were not significantly more expressed in any particular task than expected by chance and may implicate functional network components that are expressed during processes that are agnostic to task-specific mechanics, such as arousal.

### Subgraph expression adapts to transitions in cognitive demand

We next ask “How do functional subgraphs adapt to experimentally imposed changes in cognitive demand during the different cognitive control tasks?” We hypothesize that a functional subgraph is either sensitive to cognitive control processes specific to the experimentally imposed changes in cognitive demand or to the stimulus and task mechanics that are shared between low and high cognitive demand conditions of each task. To motivate our hypothesis, we examine the relative differences in the distributions of mean strength of each edge between the low demand conditions and high demand conditions of the Stroop task and the Navon task (S8 Fig), before extracting functional subgraphs using NMF. Specifically, we compare the strength of an edge during the low demand condition of a cognitive task to its strength during the high demand condition of the task by computing the mean difference of Fisher’s *r*-to-*Z* transformed correlations over subjects, separately for positive and negative correlations. For the Stroop task, we observe: (i) stronger positive correlations within the dorsal attention system and between the dorsal attention, cerebellar, default mode, and frontoparietal systems, and (ii) stronger negative correlations within the cortical limbic system and between the cortical limbic system and other broadly distributed cognitive systems during the high demand condition compared to the low demand condition. For the Navon task, we observe: (i) stronger positive correlations within and between the dorsal attention, visual, and cerebellar systems, (ii) stronger positive correlations within the frontoparietal system, and between the frontoparietal system and other broadly distributed cognitive systems, and (iii) stronger negative correlations within somatosensory and ventral attention systems, and between somatosensory and ventral attention systems and other broadly distributed cognitive systems during the high demand condition compared to the low demand condition.

**Fig 5:**
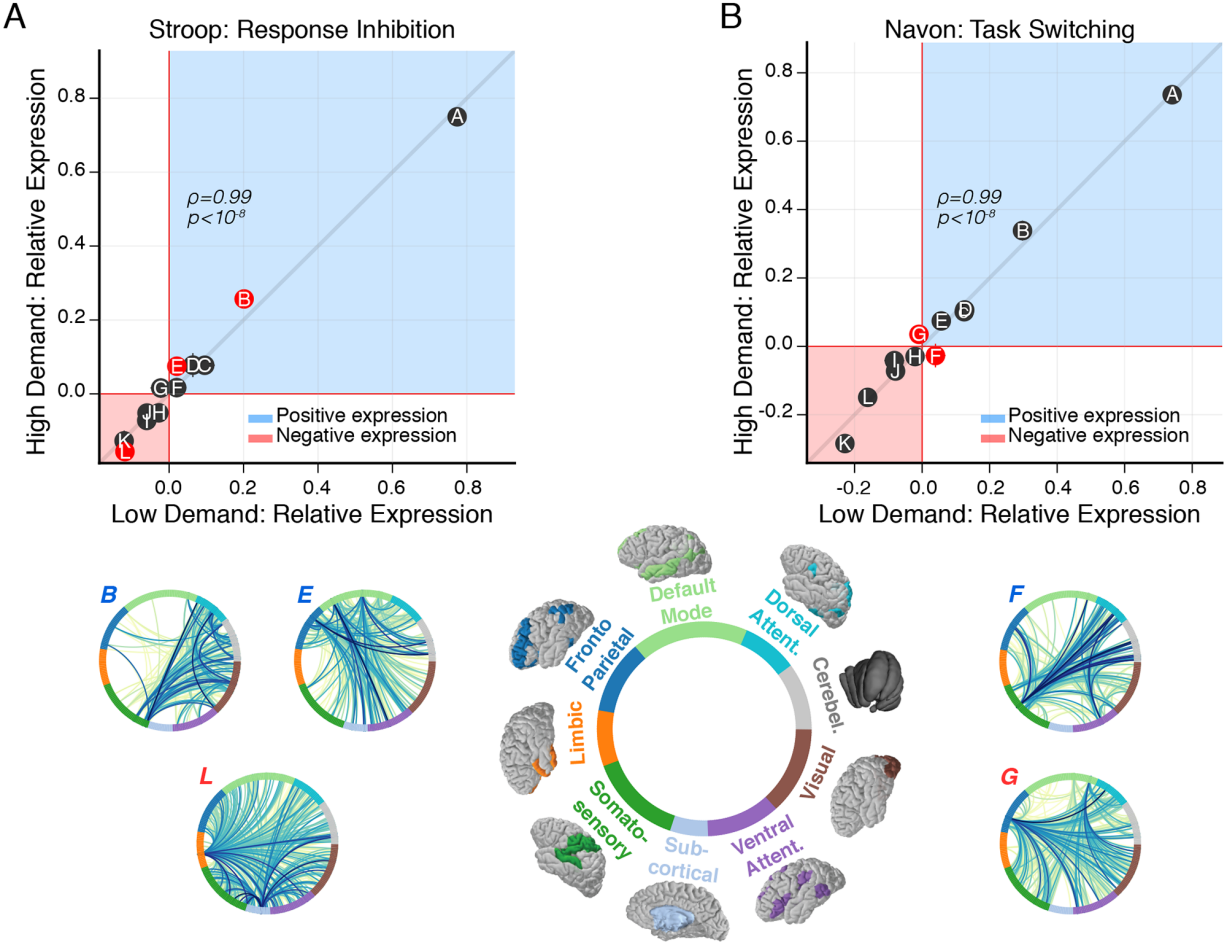
Modulation of subgraph expression coincides with increased cognitive demand. (*A*) Relationship between relative subgraph expression during the low cognitive demand condition and the high cognitive demand condition of the Stroop task. Each point represents relative subgraph expression averaged over subjects. Horizontal (vertical) error bars represent standard error of the mean for the low (high) demand condition. Similarly plotted for the low demand condition and high demand condition of the Navon task (shown in *B*). Generally, relative subgraph expression during the low demand condition is significantly associated with relative subgraph expression during the high demand condition for the Stroop task (Spearman’s *ρ*, *ρ* = 0.99, *p* = 4.1^−9^) and for the Navon task (Spearman’s *ρ*, *ρ* = 0.99, *p* = 4.1 × 10^−9^). These results imply that the rank of a subgraph in terms of its overall expression relative to other subgraphs is similar between the low cognitive demand condition and the high cognitive demand condition for the Stroop task and for the Navon task. To test whether individual subgraphs vary in the amount they are expressed as cognitive demand increases, we compare the distribution of relative subgraph expression between the low demand condition and the high demand condition for each task using paired *t*-tests and FDR correction for multiple comparisons. For the Stroop task, we find greater positive expression during the high demand condition than the low demand condition for subgraph *B* (*t*_27_ = 3.3, *p* = 2.7 × 10^−3^) and subgraph *E* (*t*_27_ = 3.2, *p* = 3.6 × 10^−3^), and we find greater negative expression during the high demand condition than the low demand condition for subgraph *L* (*t*_27_ = 2.5, *p* = 0.01). For the Navon task, we find greater positive expression during the high demand condition than the low demand condition for subgraph *G* (*t*_27_ = 2.9, *p* = 8.2 × 10^−3^), and we find greater negative expression during the high demand condition than the low demand condition for subgraph *F* (*t*_27_ = 2.7, *p* = 0.01). These results collectively suggest that subgraph expression shifts alongside changes in cognitive demand in a manner that is specific to each cognitive task. Specifically, the change in subgraph expression that accompanies an increase in cognitive demand may involve an increase in correlated or anticorrelated dynamics. These dynamics potentially implicate an antagonistic network mechanism of cognitive demand whereby one set of subgraphs engage through more positive expression while another set of subgraphs disengage through more negative expression.

We use these results to inform our expectation regarding cognitive systems that might be involved in functional subgraphs that adapt to changes in cognitive demand. We first examine the relationship between the mean relative expression of a subgraph during the low cognitive demand condition of a task and during the high cognitive demand condition of a task (Fig 5). We find that the expression of a subgraph during the low cognitive demand condition is significantly associated with its expression during the high cognitive demand condition for the Stroop task (Spearman’s *ρ*, *ρ* = 0.99, *p* = 4.1 × 10^−9^) and for the Navon task (Spearman’s *ρ*, *ρ* = 0.99, *p* = 4.1 × 10^−9^), suggesting that subgraphs follow a similar ranked order in their relative expression before and after the increase in cognitive demand. Critically, this result implies that subgraphs follow a consistent hierarchy of expression during the low demand and high demand conditions of each task.

While the relative relationships between subgraph expression are preserved between cognitive demand conditions, we also identify differences in the magnitude of subgraph expression between demand conditions. Specifically, we compare the distribution of relative subgraph expression between the low cognitive demand condition and the high cognitive demand condition of each task for each subgraph using paired *t*-tests and FDR correction for multiple comparisons. For the Stroop task, we find greater positive expression during the high demand condition than the low demand condition for subgraph *B* (*t*_27_ = 3.3, *p* = 2.7 × 10^−3^) and subgraph *E* (*t*_27_ = 3.2, *p* = 3.6 × 10^−3^) and greater negative expression during the high demand condition than the low demand condition for subgraph *L* (*t*_27_ = 2.5, *p* = 0.01). These findings suggest that the Stroop task (i) exhibits greater correlated BOLD dynamics during the high demand condition than during the low demand condition within and between dorsal attention, visual and cerebellar systems (subgraph *B*), and within and between default mode and frontoparietal systems (subgraph *E*), and (ii) exhibits greater anticorrelated BOLD dynamics during the high demand condition than during the low demand condition within the limbic and subcortical systems, and between the limic and subcortical systems and other broadly distributed cognitive systems (subgraph *L*). For the Navon task, we find greater positive expression during the high demand condition than during the low demand condition for subgraph *G* (*t*_27_ = 2.9, *p* = 8.2 × 10^−3^), and we find greater negative expression during the high demand condition than during the low demand condition for subgraph *F* (*t*_27_ = 2.7, *p* = 0.01). These findings suggest that the Navon task (i) exhibits greater correlated BOLD dynamics during the high demand condition than during the low demand condition between frontoparietal and default mode systems and other broadly distributed cognitive systems (subgraph *G*), and (ii) exhibits greater anticorrelated BOLD dynamics during the high demand condition than during the low demand condition within somatosensory and ventral attention systems, and between somatosensory and ventral attention systems and other broadly distributed cognitive systems (subgraph *F*).

Overall, we find that functional subgraphs follow a general hierarchy of expression that remains consistent between low cognitive demand conditions and high cognitive demand conditions, and adaptively shift their expression alongside experimentally invoked changes in cognitive demand. Critically, our findings imply that subgraphs may maintain a robust network representation of each cognitive control task between different states of cognitive demand and may adaptively encode different cognitive control processes via shifts in positive or negative expression such that the overall hierarchical representation of the task remains undisturbed. These shifts in subgraph expression are evidently coordinated through changes in correlated and anticorrelated BOLD dynamics involving select subgraphs. Accordingly, these results suggest that the functional brain network may utilize task-specific control strategies by coordinating antagonistic changes in the co-activation between different cognitive systems during pre-potent response inhibition (Stroop task) and during perceptual, rule-based task switching (Navon task).

### Recruitment of functional subgraphs related to task performance

**Fig 6:**
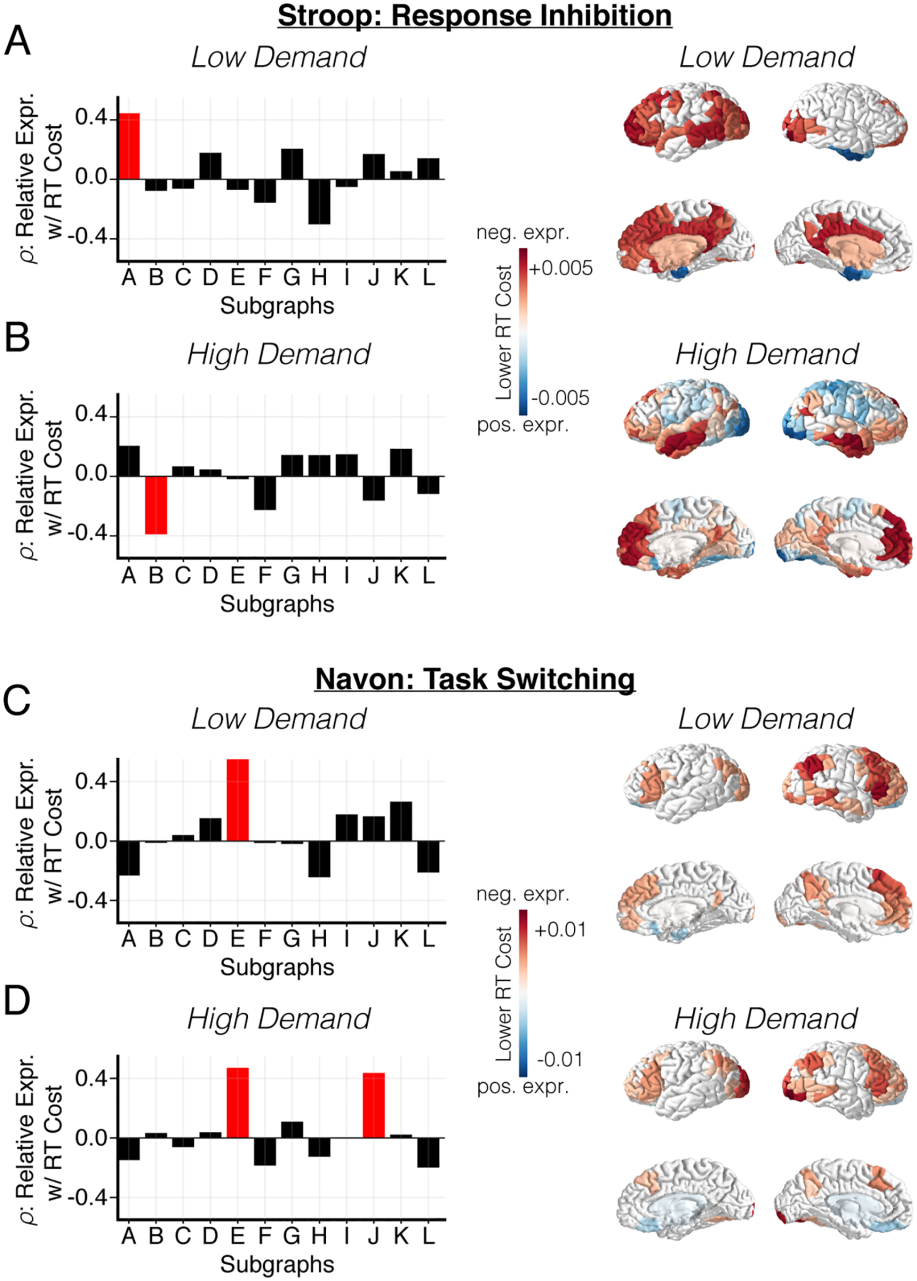
Subgraph expression stratifies inter-individual task performance. (*A-D; left*) To examine the link between subgraph dynamics and behavior, we compare subgraph expression to task-specific performance cost across individuals. Specifically, we compute the Spearman’s *ρ* between relative subgraph expression, averaged across low demand or high demand task blocks of each participant, and reaction time cost—difference between reaction time on high demand blocks and low demand blocks (lower is better)—averaged across task blocks of each participant. Values of *ρ* greater than zero imply that greater *negative* subgraph expression is associated with lower reaction time cost and values of *ρ* less than zero imply that greater *positive* subgraph expression is associated with lower reaction time cost. (*A-B; left*) Distribution of Spearman’s *ρ* between relative subgraph expression and reaction time cost for each subgraph during the low cognitive demand condition and the high cognitive demand condition of the Stroop task. Subgraphs with significant correlations are colored red (*p* < 0.05; uncorrected for multiple comparisons). (*C-D; left*) Distribution of Spearman’s *ρ* between relative subgraph expression and reaction time cost for each subgraph during the low cognitive demand condition and the high cognitive demand condition of the Navon task. Subgraphs with significant correlations are colored red (*p* < 0.05; uncorrected for multiple comparisons). (*A-D; right*) To assess which brain regions are more influential in subgraphs whose expression is associated with lower reaction time cost, we compute a participation score for each brain region by computing its node strength in each subgraph and calculating the sum of each brain region’s node strength across subgraphs, weighted by the Spearman’s *ρ* value. Intuitively, a more positive participation score implies that a brain region is more involved in subgraphs with greater negative expression in individuals with lower reaction time cost, and a more negative participation score implies that a brain region is more involved in subgraphs with greater positive expression in individuals with lower reaction time cost. We compare participation scores to a null distribution that is generated by permuting edges in the subgraph adjacency matrix 10000 times and recomputing the participation score for each permutation (*p* < 0.05; Bonferroni corrected for multiple comparisons). Brain regions with significantly positive participation scores (associated with anticorrelated dynamics) are colored in red, and brain regions with significantly negative participation scores (associated with correlated dynamics) are colored in blue.

We next examine how the recruitment of functional subgraphs relates to the change in inter-individual performance as participants invoke cognitive control mechanisms. Our approach is based upon prior studies that posit a functional role of antagonistic dynamics between correlated and anti-correlated brain activity in cognitive control processes [28, 29]. We use the subgraph characterization of the functional network to directly examine how behavioral performance is related to the extent that distinct networks exhibit more correlated or anti-correlated dynamics. To evaluate the change in an individual’s task performance—also known as performance cost—we separately compute mean change in an individual’s reaction time between consecutive blocks of the low demand condition and the high demand condition. Intuitively, a lower reaction time cost indicates better performance and a higher reaction time cost indicates worse performance. Using the reaction time cost as a behavioral marker for inter-individual differences in cognitive control processes, we study the functional role of subgraphs during the following two phases of the cognitive control tasks: (i) task activation associated with the low cognitive demand condition, and (ii) task control associated with the high cognitive demand condition. To quantify the association between subgraph expression and performance, we first compute each individual’s relative subgraph expression as the difference between the likelihood that a subgraph is positively expressed (i.e., functional dynamics are correlated) and the likelihood that a subgraph is negatively expressed (i.e., functional dynamics are anti-correlated). We next use Spearman’s *ρ* to assess the relationship between relative subgraph expression during the low or high demand condition, and the reaction time cost across individuals on each task (Fig 6A–6D; left). Indeed, if the correlation between relative expression and reaction time cost is positive, then individuals who express more correlated dynamics (and less anti-correlated dynamics) within a subgraph exhibit poorer performance and individuals who express more anti-correlated dynamics (and less correlated dynamics) within a subgraph exhibit better performance. This analysis approach enables us to understand the extent to which behavior is explained by both the degree to which regions in a subgraph engage with one another via correlated dynamics and the degree to which regions in a subgraph disengage from one another via anti-correlated dynamics.

We find a diverse set of subgraphs whose relative expression during low demand, task activation conditions or high demand, task control conditions correlate with reaction time cost. For the Stroop task, we find that a lower reaction time cost (better performance) is associated with: (i) greater negative expression of subgraph *A* (*ρ* = 0.44, *p* = 0.01; uncorrected for multiple comparisons) during the low demand condition, and (ii) greater positive expression of subgraph *B* (*ρ* = −0.39, *p* = 0.04; uncorrected for multiple comparisons) during the high demand condition. These results suggest that a smaller change in the reaction time between the low demand condition and the high demand condition of the Stroop task is associated with: (i) greater anticorrelated dynamics within dorsal attention, default mode, frontoparietal, somatosensory, ventral attention, and visual systems during task activation, and (ii) greater correlated dynamics within and between dorsal attention, visual, and cerebellar systems during task control. For the Navon task, we find that a lower reaction time cost (better performance) is associated with: (i) greater negative expression of subgraph *E* (*ρ* = 0.55, *p* = 2.2 × 10^−3^; uncorrected for multiple comparisons) during the low demand condition, and (ii) greater negative expression of subgraph *E* (*ρ* = 0.47, *p* = 0.01; uncorrected for multiple comparisons) and subgraph *J* (*ρ* = 0.44, *p* = 0.02; uncorrected for multiple comparisons) during the high demand condition. These results suggest that a smaller change in the reaction time between the low demand condition and the high demand condition of the Navon task is associated with: (i) greater anticorrelated dynamics within default mode and frontoparietal systems, and between default mode and frontoparietal systems and other broadly distributed cognitive systems during task activation, and (ii) greater anticorrelated dynamics within default mode, frontoparietal, and visual systems, and between default mode, frontoparietal, and visual systems and other broadly distributed cognitive systems during task control. In sum, we find that changes in the correlated and anticorrelated BOLD dynamics within and between distributed cognitive systems is associated with cognitive processes during task activation and task control that explain inter-individual differences in performance during cognitive control tasks. Based on these data and our previous result that subgraphs maintain a consistent hierarchical organization in terms of their ranked expression between cognitive demand conditions, our findings suggest that individual variability in behavior during cognitive control may be marked by subtle individual differences in subgraph expression amid a hierarchical order that is defined at the population level.

Lastly, we ask whether there are individual brain regions that are more likely to participate in subgraphs associated with task performance. By quantifying the extent to which brain regions participate in subgraphs, we aim to link our analysis with classical univariate approaches for examining functional brain activation during cognitive control tasks. We hypothesize that brain regions commonly associated with executive and higher cognitive functions, such as frontoparietal, default mode, attention, and salience systems are more likely to participate in subgraphs that are associated with task performance. To test this hypothesis, we computed the *performance participation score*—a nodal measure linking the participation of a node in a subgraph with the relationship between the subgraph and behavioral performance. Specifically, we first compute node participation in a subgraph as the sum of the subgraph edge weights from a node to all other nodes—yielding one node participation score for each of the 262 brain regions in each of the twelve subgraphs [16]. We next compute the sum of a node’s participation scores, weighted by the Spearman’s *ρ* value between relative subgraph expression and performance cost: that is, nodes of the same subgraph were weighted by the same *ρ* value. Intuitively, a node with positive participation score tends to become disengaged in the brain network, via anti-correlated dynamics, during better task performance and a node with negative participation score tends to become engaged in the brain network, via correlated dynamics, during better task performance. To determine whether a brain region exhibits a greater participation score than expected by chance, we construct null distributions of regional participation scores by uniformly permuting the edges of each subgraph 10000 times and recomputing the regional participation score for each permutation. We retain regional participation scores that exceeded the 95% confidence interval of the null distribution after using Bonferroni correction for multiple comparisons testing.

Using this approach, we find a broad range of brain regions that are significantly involved in correlated and anti-correlated brain activity during improved task performance (Fig 6A–6D; right). For the Stroop task we observe that individuals exhibit lower reaction time cost when (i) during the low demand condition, regions within frontoparietal, default mode, subcortical, and visual systems are more disengaged from each other and regions within the cortical limbic system are more engaged with each other (Fig 6A; right), and (ii) during the high demand condition, regions within default mode and limbic systems are more disengaged from each other and regions within visual and somatosensory systems are more engaged with each other (Fig 6B; right). For the Navon task we observe that individuals exhibit lower reaction time cost when (i) during the low demand condition, regions within frontoparietal, default mode, and visual systems are more disengaged from each other and regions within the cortical limbic system are more engaged with each other (Fig 6C; right), and (ii) during the high demand condition, regions within frontoparietal, default mode, and visual systems are more disengaged with each other and regions within subcortical and limbic systems are more engaged with each other (Fig 6D; right).

Together, these results demonstrate that brain regions classically considered as key components of executive and higher cognitive functions, such as regions in frontoparietal and default mode systems, tend to be more influential in subgraphs that are associated with task performance. Notably, our approach characterizes the functional role that these brain areas play during task activation and task control based on their participation in correlated and anticorrelated BOLD dynamics. During different phases of cognitive control, regions involved in correlated dynamics may serve as integrators of task-relevant information while regions involved in anticorrelated dynamics may serve as segregators of task-relevant information.

## Discussion

In this work, we ask “What functional constraints shape internally-guided transitions in brain state during cognitive control?” To answer this question, we apply a powerful machine-learning approach referred to as non-negative matrix factorization, to dynamic functional brain networks measured during two cognitive control tasks—yielding subgraphs or clusters of temporally co-varying functional interactions between brain regions. We study the expression of these functional subgraphs during correlated and anticorrelated BOLD dynamics as subjects transition between different levels of task-induced cognitive demand. We show that the subgraphs differentiate clusters of functional interactions that are specific to the mechanics of the cognitive control tasks from those that are generalized to the network processes common to the cognitive control tasks. Specifically, we demonstrate for the first time clear evidence that functional subgraphs adaptively alter their expression depending on the type of cognitive control task and the amount of cognitive demand imposed on the system. Our results significantly extend our understanding of how objectively-defined clusters of functional interactions, beyond individual region-region co-activation, relate to transitions between cognitive states.

### Encoding dynamical rules for cognitive control

Our non-negative matrix factorization (NMF) approach enables us to objectively account for: (i) the dissociability of brain networks into composite subgraphs that are associated with specific cognitive control functions, and (ii) the flexible and adaptive expression of these putative cognitive sub-networks during fluctuations in cognitive demand. Intuitively, these subgraphs represent clusters of functional interactions whose weights tend to fluctuate together across tasks and across conditions. Unlike other graph partitioning techniques, such as community detection, that pursue a hard partitioning of network nodes into discrete clusters, NMF enables a soft partitioning of the high dimensional set of network edges into subgraphs that allow an edge to participate in multiple network sub-units [16, 22]. This capability is advantageous for examining how pairs of brain areas functionally interact within different topological contexts. Mathematically, NMF recovers a non-orthogonal spanning set of graph edges whose linear combination—weighted by dynamic expression coefficients—can reconstruct the original space of observed network topologies across the experimental task conditions. In other words, subgraphs represent a set of functional relationships for the cognitive control data from which they were recovered and subgraph expression coefficients represent the encoding of those relationships for the different task conditions (we refer the reader to [40] for a discussion on neural coding theory).

Thus from the perspective of network-based encoding of cognitive control tasks, indeed, we find that subgraphs are comprised of functional interactions that are either sensitive to the specific needs of a particular task or generalized to needs common across tasks. These data support the theory that there exist separate task-specific and task-general network architectures [30]. We examine the particular cognitive systems involved in task-specific and task-general subgraphs and find a dual-role for correlated and anticorrelated interactions between traditional cognitive control systems and the default mode system: these systems are positively expressed during cognitive control involving Stroop-based response inhibition and negatively expressed during cognitive control involving Navon-based task switching. Our finding of anticorrelated interactions between cognitive control and default mode systems is well supported by the popular theory that the task-negative, default mode system deactivates as task-positive, executive areas activate [41–43]. On the other hand, our finding of correlated interactions between these systems challenges the notion that these systems must decouple during cognitive control. Prior studies have in fact demonstrated that individuals that exhibit greater integration between the default mode network and executive areas tend to display better behavioral performance during cognitive control tasks that involve switching between task-rules [28, 44]. Based on these results, we posit that differences in the nature of functional interactions between these systems might be explained by task-specific requirements for cognitive control. Importantly, NMF demonstrates the ability to tease apart functional interactions underlying intrinsic differences in cognitive control processes by recovering task-specific subgraphs.

There is a longstanding question in network neuroscience regarding the putative roles of task-specific functional architectures and their relationship to intrinsic functional networks that generalize across tasks [30]. The canonical model posits that task-general processes shape intrinsic functional networks and task-specific processes update subsets of these intrinsic functional connections [30]. A critical assumption has been that networks related to task-specific processes also facilitate behavioral performance of the task. In this study, we present data that support the canonical model yet challenge the assumption that task processes and behavioral metrics of performance on the task stem from the same network structures. First, we find that a robust hierarchy of subgraphs persist between different forms of cognitive control processes (Fig 4A) and different levels of cognitive demand (Fig 5A and 5B). Indeed, changes in subgraph expression within the bounds of this hierarchy accompany specific task states, however we also find that the subgraphs that best predict individual differences in behavior are not necessarily those that are modulated by different task conditions. In other words, functional architectures most strongly associated with behavior may represent task-general cognitive functions that are distinct from networks that are differentially expressed between cognitive conditions, consistently across individuals. For example, the Stroop task is designed to recruit general processes related to stimulus perception and color-word discrimination as well as cognitive control processes such as inhibition of the prepotent response to an incongruent stimulus and Navon task is designed to recruit general processes such as perceptual decision making or specific processes such as decision making based on rules that periodically switch. A task-general subgraph that is modulated by lower level perceptual or cognitive processes during low and high task conditions may still be modulated differently across individuals and reflect differences in behavior. Conversely, based on recent work demonstrating that different components of functional brain networks may be highly similar or highly dissimilar across individuals [45], a task-general subgraph that does not strongly vary with individual differences in behavior might reflect intermediate task processes that are common to the low and high cognitive demand conditions. Indeed, a future study that uses NMF in conjunction with faster imaging modalities may amenably tease apart subgraphs involved with different temporal phases of cognitive control processes.

### Antagonistic push-pull control of cognitive demand

A growing body of literature in network neuroscience has shown that the brain possesses an ability to maintain a homeostasis of its own internal dynamics through antagonistic, push-pull interactions in various areas of healthy cognition [28, 29, 46] and disease [47]. Simply, push-pull control strategies may prevent imbalances of activity in complex, interconnected systems like the brain [48, 49]. A push-pull mechanism would be a critical component of cognitive control in which brain networks must perform two antagonistic functions: (i) segregated information processing in functionally-specific domains, and (ii) integrated information processing to adapt to environmentally-driven changes in cognitive demand [26]. Our results pertaining to the adaptive shifting in subgraph expression during changes in cognitive demand may be associated with a putative push-pull control mechanism in which: (i) subgraphs first establish a consistent hierarchy of expression that enforces a baseline level of expression that remains consistent relative to other subgraphs during cognitive control processes, and (ii) subgraphs then shift their expression above or below their baseline—via changes in correlated or anticorrelated BOLD dynamics—depending on cognitive demand. We posit that a push-pull mechanism might internally regulate the direction of change in subgraph expression, collectively across the network: an excessive increase or decrease in subgraph expression might disrupt the hierarchical order of subgraph expression and lead to brain states in which information is overly integrated or overly segregated across the network. In our analysis, we observe that the shift between cognitively demanding brain states involves a change in the interacting roles between brain areas distributed across several cognitive systems: including frontoparietal, default mode, attentional, and cerebellar regions. Recent studies focusing on functional interactions between cerebellum and traditional cognitive control regions [12] have suggested that the cerebellum may subserve cognitive processes related to error correction [50, 51]. Our results add new insight to this discussion by demonstrating in two different cognitive control tasks that frontoparietal, cerebellar, and sensory systems are involved in subgraphs that significantly change in expression with increasing cognitive demand.

We also consider the possibility that regulatory mechanisms involved in cognitive control might also explain differences in individual performance on cognitively demanding tasks. We found that subgraphs may be heterogeneously associated with individual cognitive performance: greater correlated BOLD dynamics and greater anticorrelated BOLD dynamics between regions of subgraphs are associated with improved task performance. These data suggest that cognitive control is associated with enhanced integration and segregation of task-relevant information between different composite sets of brain regions. In addition, we use functional subgraphs to uncover the relationship between functional interactions and sub-processes of cognitive control that differentially contribute to the performance cost associated with an increase in cognitive demand. Namely, we find subgraphs whose expression during task activation is associated with lower performance cost accompanying an increase in cognitive demand, and we find subgraphs whose expression during task control is associated with lower performance cost accompanying an increase in cognitive demand. We speculate that the rich distribution of performance modes exhibited by functional subgraphs implicates a network homeostasis on cognitive control processes [46].

Critically, we contextualize the relationship between network reorganization during task states and its relationship with task performance via the following sequence of events. First, global network correlations decrease between the fixation period and the task. As the network becomes less correlated, select subgraphs become increasingly specialized and exhibit heightened levels of expression relative to non-task related subgraphs. These task-related subgraphs remain highly expressed across individuals and inter-individual differences in expression scale with task performance. Brain regions with greatest levels of participation within task-related subgraphs are putative mediators of the relationship between subgraph expression and performance.

### Conclusions and future directions

In sum, we demonstrate that functional brain networks capably adapt their topological architecture in response to task-driven modulation in cognitive demand. Critically, we observe that cognitive control may not necessarily activate discrete cognitive brain systems, but rather recruit several interconnected systems, in concert, between changes in cognitively demanding brain states. When individuals under- or over-express functional interactions between these cognitive systems they tend to respond more slowly during difficult cognitive tasks, implicating specific brain sub-networks in facilitating or impeding an individual’s ability to transition between states.

While we narratively describe cognitive control to be recruited continuously in response to task demands, it is also important to acknowledge that cognitive control functions can be considered to be distinct processes [52] with partially dissociable substrates [53]. Given these broader debates about shared and unique CC mechanisms, we should continue to consider the spatiotemporal signatures of brain activity that distinguish between accounts of CC. Future studies could use NMF-based subgraph analysis to dissect networks involved in tasks where demand is parametrically varied and test whether a continuous or discrete representation of specific CC functions better describes observed network dynamics.

Lastly, we focus on the mechanistic role that functional brain networks play in regulating internal dynamics during cognitive control. Our novel approach and findings open new doors for querying how such regulatory mechanisms could be modulated to influence behavior. For instance, can we perturb specific network components to improve the likelihood that an individual is able to access shorter trajectories to switch between low demanding states and high demanding states? By marrying machine-learning approaches that objectively tease apart concurrent network processes attributed to different facets of cognition with burgeoning neurotechnologies such as neurofeedback [54], neurostimulation [55], or pharmacological intervention [56–58] that can exogenously control network dynamics, we can explore how disrupting network components that exhibit task-based adaptation causally influence behavior. The prospect of such scientific inquiry is equally exciting in diseases such as schizophrenia in which patients experience more probable transitions to more disruptive cognitive states.

## Materials and methods

### Study cohort

#### Ethics statement

All subjects volunteered with informed consent in writing in accordance with the Institutional Review Board/Human Subjects Committee at the University of Pennsylvania.

#### Patient demographics

A total of 30 subjects were recruited. All subjects were screened for prior history of psychiatric or neurological illness. One subject was excluded due to near-chance performance on the task (accuracy = 52%). One additional subject was excluded due to technical problems on the day of scanning. The final sample included 28 individuals (mean age = 25.6±3.5, 70% caucasian, 13 females).

### Cognitive control tasks

All participants completed a Stroop task with color-word pairings that were eligible and ineligible to elicit interference effects [24], and a local-global perception task based on classical Navon figures [25]. For the Stroop task, trials were comprised of words presented one at a time at the center of the screen printed in one of four colors—red, green, yellow, or blue -– on a gray background. For all trials, subjects responded using their right hand with a four-button response box. All subjects were trained on the task outside the scanner until proficient at reporting responses using a fixed mapping between the color and button presses (i.e., index finger = “red”, middle finger = “green”, ring finger = “yellow”, pinky finger = “blue”). Trials were presented in randomly intermixed blocks containing trials that were either eligible or ineligible to produce color-word interference effects. In the scanner, blocks were administered with 20 trials apiece separated by 20 s fixation periods with a black crosshair at the center of the screen. Each trial was presented for a fixed duration of 1900 ms separated by an interstimulus interval of 100 ms during which a gray screen was presented. In the trials ineligible for interference, the words were selected to not conflict with printed colors (“far,” “horse,” “deal,” and “plenty”). In the trials eligible for interference (i.e., those designed to elicit the classic Stroop effect [24]), the words were selected to introduce conflict (i.e., printed words were “red,” “green,” “yellow,” and “blue” and always printed in an incongruent color). In our analysis, we refer to blocks that are eligible (ineligible) to produce color-word interference effects as *high demand* (*low demand*) conditions (Fig 1B).

For the Navon task, local-global stimuli were comprised of four shapes—a circle, X, triangle, or square—that were used to build the global and local aspects of the stimuli. On all trials, the local feature did not match the global feature, ensuring that subjects could not use information about one scale to infer information about another. Stimuli were presented on a black background in a block design with three blocks. In the first block type, subjects viewed white local-global stimuli. In the second block type, subjects viewed green local-global stimuli. In the third block type, stimuli switched between white and green across trials uniformly at random with the constraint that 70% of trials included a switch in each block. In all blocks, subjects were instructed to report only the local features of the stimuli if the stimulus was white, and to report only the global feature of the stimuli if the stimulus was green. Blocks were administered in a random order. Subjects responded using their right hand with a four-button response box. All subjects were trained on the task outside the scanner until proficient at reporting responses using a fixed mapping between the shape and the button presses (i.e., index finger = “circle”, middle finger = “X”, ring finger = “triangle”, and pinky finger = “square”). In the scanner, blocks were administered with 20 trials apiece separated by 20 s fixation periods with a white crosshair at the center of the screen. Each trial was presented for a fixed duration of 1900 ms separated by an interstimulus interval of 100 ms during which a black screen was presented. In our analysis, we refer to blocks that switch between local-global perception as the *high demand* condition and blocks that do not switch as the *low demand* condition (Fig 1C).

### Data acquisition and pre-processing

We acquired T1-weighted anatomical scans on a Siemens 3.0T Tim Trio for all subjects. Anatomical scans were segmented using FreeSurfer [59] and parcellated using the connectome mapping toolkit [31] into *N* = 234 cortical and subcortical brain regions. We also included a cerebellar parcellation (*N* = 28 brain regions [32]) by using FSL to nonlinearly register the individual’s T1 to MNI space. Then, we used the inverse warp parameters to warp the cerebellum atlas to the individual T1. Finally, we merged the cerebellar label image with the dilated cortical and subcortical parcellation image resulting in *N* = 262 brain regions.

Functional magnetic resonance imaging data was acquired on a 3.0T Siemens Tim Trio whole-body scanner with a whole-head elliptical coil by means of a single-shot gradient-echo T2* (TR = 1500 ms; TE = 30 ms; flip angle = 60 degrees; FOV = 19.2 cm, resolution 3mm x 3mm x 3mm). Preprocessing was performed using FEAT v. 6.0 (fMRI Expert Analysis Tool) a component of the FSL software package [60]. To prepare the functional images for analyses, we completed the following steps: skull-stripping with BET to remove non-brain material, motion correction with MCFLIRT (FMRIB’s Linear Image Registration Tool; [60]), slice timing correction (interleaved), spatial smoothing with a 6-mm 3D Gaussian kernel, and high pass temporal filtering to reduce low frequency artifacts. We also performed EPI unwarping with fieldmaps to improve subject registration to standard space. Native image transformation to a standard template was completed using FSL’s affine registration tool, FLIRT [60]. Subject-specific functional images were co-registered to their corresponding high-resolution anatomical images via a Boundary Based Registration technique (BBR [61]) and were then registered to the standard MNI-152 structural template via a 12-parameter linear transformation. Finally, each participant’s individual anatomical image was segmented into grey matter, white matter, and CSF using the binary segmentation function of FAST v. 4.0 (FMRIB’s Automated Segmentation Tool [62]). The white matter and CSF masks for each participant were then transformed to native functional space and the average timeseries were extracted. Based on the commonly accepted notion that smoothing reduces scan-related, spatially-distributed Gaussian noise across voxels and enhances BOLD signal-to-noise ratio, we conducted smoothing by applying a kernel with full-width half-maximum of 6 mm to voxels prior to ROI time series extraction. An important consideration of smoothing is that voxels at the edge of an ROI may contain overlapping information from adjacent ROIs. However, our analysis occurs at the level of the aggregate BOLD activity across many voxels in an ROI, and thus voxel-level precision was not a goal in this study. The white matter and CSF signals were used as confound regressors on the time series along with 18 translation and rotation parameters as estimated by MCFLIRT [63]. To preserve natural anti-correlation in the BOLD signal, we did not regress the global signal [64].

We refer the reader to [65] for additional methodological details regarding data acquisition and pre-processing.

### Constructing functional brain networks

We constructed functional brain networks to study the functional interactions between brain regions during the Stroop and Navon cognitive control tasks. To measure functional interactions, we first separately divided the BOLD signal into six low demand blocks, six high demand blocks, and twelve fixation blocks (before each cognitive demand block) for each behavioral task of each subject. Each block contained 20 samples or 30 seconds of signals (Fig 2B). We next computed a Pearson correlation coefficient between each pair of BOLD signals from the *N* brain regions (graph nodes) in each of the *K* experimental blocks. We then aggregated correlations (graph edges) into an *N* × *N* × *K* adjacency matrix **A** for each subject. We note that due to confounding delays in hemodynamic response, it is possible that fixation blocks contain both task-related and task-unrelated activity. To mitigate this concern, we take two steps. First, we align each block with the peak hemodynamic response by shifting analysis windows by 4 TRs, which corresponds to the canonical hemodynamic lag of 6 seconds. Second, we compute NMF-based subgraphs (see next section) using fixation blocks to increase the length of the physiologic signal, but we restrict our analysis of the subgraphs specifically to task blocks.

To analyze positively correlated (correlated) and negatively correlated (anticorrelated) functional interactions, we separated positively-weighted edges from negatively-weighted edges for each block *k* in **A** using a threshold of zero. This procedure resulted in a thresholded adjacency matrix A* of size *N* × *N* × 2 × *K* where each block *k* is associated with one *N* × *N* matrix with positive edge weights and another *N* × *N* matrix with negative edge weights (Fig 2C). We retain all correlation values after the thresholding procedure such that both positive adjacency matrices and negative adjacency matrices are both fully-weighted graphs.

An alternate representation of the adjacency matrix A* is a two-dimensional network configuration matrix $\hat{A}*$, which tabulates all *N* × *N* pairwise edge weights across *K* blocks, and across positive and negative edge types (Fig 2D). Due to symmetry of $A_{k}^{*}$, we unravel the upper triangle of $A_{k}^{*}$, resulting in the weights of *N*(*N* − 1)/2 connections. Thus, $\hat{A}*$ has dimensions *N*(*N* − 1)/2 × 2*K.

### Clustering functional networks into subgraphs

To identify network subgraphs—sets of network edges whose strengths co-vary over experimental task conditions—we applied an unsupervised machine learning algorithm called non-negative matrix factorization (NMF) [21] to the network configuration matrix. This technique enabled us to pursue a parts-based decomposition of the network configuration matrix into subgraphs with expression coefficients that vary with time (Fig 2E and 2F). Briefly, NMF holds two distinct advantages to principal components analysis (PCA) and independent components analysis (ICA) for studying components of interconnected network structures. First, PCA/ICA quantify subgraphs that are statistically orthogonal/independent from each other, while NMF quantifies subgraphs that are statistically redundant such that they can flexibly co-occur with other subgraphs during different brain states. The unique property of NMF to characterize overlapping network structures is conceptually valuable for the analysis of brain graphs, which assume that each node encompasses statistical relationships with all other nodes in the network—this assumption is violated by PCA/ICA. Second, PCA/ICA arbitrarily assign positive and negative weights to subgraphs, while NMF enforces non-negative weights to subgraphs. The non-negative property of NMF uniquely quantifies subgraphs that are additive parts of the network and interpretable on the basis of their positive contribution to the functional network at each point in time—this interpretation is obfuscated by PCA/ICA. For further, in-depth discussion regarding network subgraphs, we refer the reader to [16]. For recent applications of NMF to the study of functional brain networks, please see [22, 23, 66, 67].

To apply NMF to functional networks, we first computed the magnitude of the network configuration matrix $\hat{A}*$ such that all entries of the matrix were non-negative. We next applied two normalization procedures to account for differences in the magnitude of edge weights between positive correlations and negative correlations and between study participants. First, based on the finding that mean negative correlations are significantly lower in magnitude than mean positive correlations across subjects (paired *t*-test; *t*_27_ = 20.0, *p* = 9.7 × 10^−18^; S1E Fig), we sought to normalize the distribution of positive edge weights and negative edge weights for each observed graph (each row of the configuration matrix). Therefore, we divided the edge weights in each row of the configuration matrix by their sum such that the weight edge density for each observed graph was equal to one. Second, based on the finding that the distribution of edge weights differs between subjects (one-way ANOVA; *F* = 2.5, *p* = 3.5 × 10^−5^; S1A Fig), we sought to standardize the vector of weights associated with each edge (each column of the configuration matrix), separately, for each subject. Therefore, we scaled the weights of each edge by their Euclidean length (L2-norm), separately, for each subject [68]. We also note that BOLD autocorrelation was not removed from the measured edge weights. As NMF is a linear operation and based on a recent study showing that the edge weights before removing BOLD autocorrelation are linearly correlated with edge weights after removing BOLD autocorrelation [69], we did not expect this procedure to influence NMF analysis.

We next formulated the matrix factorization problem $\hat{A}*\approx WH\;\text{s}.\text{t}.\;W>=0,\;H>=0$ as the decomposition of the network configuration matrix $\hat{A}*$ into two non-negative matrices **W**—the weighted subgraph matrix consisting of recurring patterns of functional interactions, or network edges—and **H**—the dynamic expression matrix consisting of coefficients reflecting the weight of a subgraph during different task conditions of each subject [16]. To quantify **W** and **H**, we optimized the following cost function:

$$\begin{array}{c} {\text{min}}_{W,H}\frac{1}{2}{\left\|\hat{A}-WH\right\|}_{F}^{2}+\alpha {\left\|W\right\|}_{F}^{2}+\beta \sum_{t=1}^{T}{\left\|H\left(:,t\right)\right\|}_{1}^{2}, \end{array}$$ (1)

where *m* ∈ [2, min(*N*(*N* − 1)/2, *T*) − 1] is the number of subgraphs to decompose, *β* is a penalty weight to impose sparse temporal expression coefficients, and *α* is a regularization of the interaction strengths for subgraphs [70]. To solve the NMF equation, we used an alternating non-negative least squares with block-pivoting method with 100 iterations for fast and efficient factorization of large matrices [71]. We initialized **W** and **H** with non-negative weights drawn from a uniform random distribution on the interval [0, 1].

To select the parameters *m*, *β*, and *α*, we pursued a random sampling scheme—shown to be effective in optimizing high-dimensional parameter spaces [16, 72]—in which we re-ran the NMF algorithm for 1000 parameter sets in which *m* is drawn from U(3,50), *β* is drawn from U(0.01,5), and *α* is drawn from U(0.01,5) (S2 Fig). We evaluated subgraph learning performance based on a four-fold cross-validation scheme in which the twenty eight subjects are uniformly partitioned into folds of seven subjects and, iteratively, three folds are used to identify subgraphs and the held-out fold is used to compute the cross-validation error (${\left\|\hat{A}-WH\right\|}_{F}^{2}$). The optimal parameter set should yield subgraphs that minimize the cross-validation error and reliably span the space of observed network topologies [16]. Based on these criteria, we identified an optimum parameter set $\left(\bar{m},\bar{\beta },\bar{\alpha }\right)$ that exhibited a low residual error in the bottom 5^th^ percentile of our random sampling scheme (S2G and S2I Fig).

Due to the non-deterministic nature of this approach, we integrated subgraph estimates over multiple runs of the algorithm using *consensus clustering*—a general method of testing robustness and stability of clusters over many runs of one or more non-deterministic clustering algorithms [73]. Our adapted consensus clustering procedure entailed the following steps: (i) run the NMF algorithm *R* times per network configuration matrix, (ii) concatenate subgraph matrix **W** across *R* runs into an aggregate matrix with dimensions $E\times (R*\bar{m})$, and (iii) apply NMF to the aggregate matrix to determine a final set of subgraphs **W**_consensus_ and expression coefficients **H**_consensus_ (we refer the reader to [16] for more details). In this study, we set *R* = 1000.

### Subgraph core-periphery index

To investigate putative core-periphery organization in each functional subgraph, we quantify the core-periphery index as a measure of the balance between mean edge strength within each cognitive system and mean edge strength of each cognitive system to all other cognitive systems. Specifically, we define the core-periphery index for a symmetric, subgraph adjacency matrix W* with dimensions *N* × *N* using the following equations:

$$\begin{array}{c} {\text{core}}_{s}=\frac{1}{\left|s\right|}\sum_{ij}\left[W_{ij}^{*}\right]\delta \left(s_{i},s_{j}\right) \end{array}$$ (2)

$$\begin{array}{c} {\text{periphery}}_{s}=\frac{1}{\left|s|*(N-|s|\right)}\sum_{ij}\left[W_{ij}^{*}\right]\left(1-\delta \left(s_{i},s_{j}\right)\right) \end{array}$$ (3)

$$\begin{array}{c} \text{core-periphery}=\frac{1}{9}\sum_{s=1}^{9}\frac{core_{s}-periphery_{s}}{core_{s}+periphery_{s}} \end{array}$$ (4)

where *N* is the number of network regions, *s* is one of nine cognitive systems, |*s*| is the number of nodes in cognitive system *s*, *s*_*i*_, *s*_*j*_ refer to the cognitive system assignments of nodes *i* and *j*, and *δ*(*s*_*i*_, *s*_*j*_) = 1 if *s*_*i*_ = *s*_*j*_ and *δ*(*s*_*i*_, *s*_*j*_) = 0 if *s*_*i*_ ≠ *s*_*j*_. Intuitively, the core-periphery index is bounded between −1 and +1, where positive values indicate greater subgraph edge strength within a cognitive system, indicating that the subgraph reflects functional interactions within a network core, negative values indicate greater subgraph edge strength between cognitive systems, indicating that the subgraph reflects functional interactions within a network periphery, values approaching zero imply that a subgraph reflects balanced functional interactions between the network core and the network periphery. To examine the specific cognitive systems that participate in core-periphery organization of each subgraph, we first generate 10000 surrogates of each subgraph by randomly permuting subgraph edges to disrupt system-level architecture. We compute the core score and the periphery score for each cognitive system *s* of each of the surrogates, separately for each subgraph. Using Bonferroni correction for multiple comparisons testing, we identify subgraph-specific cognitive systems that exhibit significantly greater core and periphery scores than expected by the surrogate model S6 Fig.

### Test-retest reliability of subgraphs

It is important to consider the reproducibility of subgraphs measured using NMF given different data splits. To quantify the reproducibility of functional subgraphs, we measured the extent to which the pattern of subgraph edge weights measured in one dataset predicts the pattern of subgraph edge weights measured in a second dataset. Specifically, we first divided the whole cognitive control dataset into two datasets such that the first dataset contains the first three experimental blocks across subjects and the second dataset contains the second three experimental blocks across subjects. We next applied NMF using the optimal parameter set to the two datasets (${\hat{A}}_{1}$ corresponds to the network configuration matrix of the first dataset and ${\hat{A}}_{2}$ corresponds to the network configuration matrix of the second dataset), resulting in two subgraph matrices (**W**_1_ and **W**_2_). Note that the subgraphs along the columns of **W**_1_ may not necessarily be ordered similarly as the subgraphs along the columns of **W**_2_ due to the stochastic nature of the NMF algorithm. To reorder subgraphs from the second dataset such that they correspond to the same order as subgraphs from the first dataset, we sought a mapping *X*_*i*,*j*_ of subgraph $W_{1}^{i}$ to subgraph $W_{2}^{j}$, where *X* is a Boolean matrix that prescribes whether the *i*^th^ subgraph from the first dataset is uniquely assigned to the *j*^th^ subgraph from the second dataset. The cost *C*_*i*,*j*_ associated with assigning $W_{1}^{i}$ to $W_{2}^{j}$ is equal to $\left\|W_{1}^{i}-W_{2}^{j}\right\|$. To determine a unique *X*, we minimized the cost function ∑_*i*_∑_*j*_ *C*_*i*, *j*_ *X*_*i*, *j*_ using the well-known Hungarian algorithm [74]. After calculating an optimal assignment between subgraphs of the two datasets, we measured the similarity in the pattern of edge weights between assigned subgraph pairs (*i*, *j*) by computing the Pearson correlation coefficient. We compared the true Pearson correlation coefficient of every subgraph pair to a null distribution in which we re-computed the Pearson correlation coefficient between every possible, non-assigned subgraph pair. This approach enabled us to assess the reproducibility of each individual subgraph based on the magnitude of the Pearson correlation similarity measure relative to that expected by chance.

## Supporting information

S1 Fig Distribution of edge weights in the dynamic functional network. (*A*) Distribution of edge weights across all experimental blocks for each of the 28 participants in the study. We find a significant difference in the edge weights between subjects (one-way ANOVA; *F* = 2.5, *p* = 3.5 × 10^−5^). (*B*) Distribution of mean edge weight across subjects for each cognitive control task. We find no significant difference in mean edge strength across subjects between blocks during the Stroop task and blocks during the Navon task (paired *t*-test, *t*_27_ = −1.5, *p* = 0.14). (*C*) Distribution of mean edge weight across subjects for fixation blocks and task blocks. We find a significant decrease in mean edge strength across subjects between blocks during the fixation period and blocks during the cognitive control task period (paired *t*-test, *t*_27_ = 4.7, *p*6.3 × 10^−5^). (*D*) Distribution of mean edge weight across subjects for low demand blocks and for high demand blocks. We find no significant difference in mean edge strength across subjects between blocks during the low cognitive demand conditions and blocks during the high cognitive demand conditions (paired *t*-test, *t*_27_ = 0.35, *p* = 0.73). (*E*) Distribution of mean magnitude edge weight for positive correlations and negative correlations across subjects. We find that the magnitude of negative edge weights is significantly lower than the magnitude of positive edge weights (paired *t*-test; *t*_27_ = 20.0, *p* = 9.7 × 10^−18^).
(TIF)

S2 Fig Parameter optimization for non-negative matrix factorization. (*A-C*) NMF-based subgraph detection requires optimizing three parameters: the number of subgraphs *m*, the sparsity of subgraph edge weights *β*, and the regularization of temporal expression coefficients *α*. To characterize this parameter space, we randomly sampled *m*, *β*, and *α* from a three-dimensional uniform distribution (*m* ∈ [3, 50], *β* ∈ [0.01, 5.0], *α* ∈ [0.01, 5.0]) and applied NMF to the configuration matrix using each parameter set. Kernel density estimate of each bivariate distribution is indicated by the contour plot, where darker shades of blue indicate greater probability mass of the random sampling distribution. Optimal parameters are the average parameter values that yielded cross-validation error in the bottom 5% of the sampling distribution and are indicated by the dashed orange line.
(TIF)

S3 Fig Relationship between motion confound and subgraph expression. We test whether a functional subgraph is confounded by motion artifact by estimating the correlation between the mean subgraph expression and the mean motion score over subjects. Using a Spearman’s *ρ* and FDR correction for multiple comparisons, we find that the expression of subgraph *B* decreases with increasing motion (*ρ* = −0.53, *p* = 3.2 × 10^−3^).
(TIF)

S4 Fig Subgraph reproducibility between split experimental blocks. Test-retest reliability of subgraphs decomposed from the first three experimental blocks and second three experimental blocks of the cognitive control dataset. NMF was separately applied to each split dataset to identify two sets of subgraphs. Subgraphs were uniquely paired between the two datasets using the Hungarian assignment algorithm [74] and the minimum Euclidean distance between subgraph edge vectors as a measure of assignment cost. To quantify subgraph reliability, we computed the Pearson correlation between pairs of assigned subgraphs and ranked subgraph pairs in decreasing order of correlation. We generated a null distribution of correlations between all possible non-assigned subgraph pairs (indicated by red dashed lines at the 95% confidence interval). Subgraphs with significantly greater correlation than expected by the null distribution are colored blue (*p* < 0.05; Bonferroni corrected for multiple comparisons). We found eleven of twelve subgraph pairs were more reliable than expected by chance assignment across the split dataset.
(TIF)

S5 Fig Adjacency matrix representation of functional subgraphs. We visualized the edge weights associated with a functional subgraph as a symmetric and fully-weighted adjacency matrix with size 262 × 262, where 262 is the number of nodes in the functional network. Based on the assignment of each of the 262 brain regions into one of nine putative cognitive systems [33]—dorsal attention (DAN), default mode (DMN), frontoparietal (FPN), limbic (LIM), somatosensory (SMN), subcortical (SUB), ventral attention (VAN), visual (VIS), and cerebellum (CRB)—we reorganize the rows and columns of each adjacency matrix such that nodes assigned to the same cognitive system are contiguously ordered. We observe subgraphs whose strongest edges (lighter shades of purple) tend to fall within well-defined boundaries of known cognitive systems.
(TIF)

S6 Fig Functional subgraphs capture distributed interactions between cognitive systems. We determined whether functional subgraphs reflect functional interactions within and between known cognitive brain systems using a previously documented approach [22]. Based on the assignment of each of the 262 brain regions into one of nine putative cognitive systems [33]—dorsal attention (DAN), default mode (DMN), frontoparietal (FPN), limbic (LIM), somatosensory (SMN), subcortical (SUB), ventral attention (VAN), visual (VIS), and cerebellum (CRB)—we computed the mean subgraph edge weight between brain regions within the same cognitive system (within-system edge weight) and mean subgraph edge weight between brain regions of one system to brain regions in all other systems (between-system edge weight). Here, we plot the mean within-system edge weight and the mean between-system edge weight for each cognitive system and each functional subgraph as horizontal bars; the mid-line implies a mean edge weight of zero, bars to the left of the midline correspond to mean between-system edge weight, and bars to the right of the midline correspond to mean within-system edge weight. Error bars correspond to standard error of the mean edge weight. To assess whether a within-system or between-system edge weight was more likely observed due to the topology of the subgraph than expected by chance, we generated a null distribution for each system-level interaction for each subgraph by permuting a subgraph’s edge weights between nodes 10000 times and recomputing the average edge weight for each permutation. We then compared each true mean edge weight to the null distribution (shaded in gray) and retained only significant within-system and between-system edge weights (*p* < 0.05; Bonferroni corrected for multiple comparisons). Cognitive systems with significant within-system edge weight or significant between-system edge weight are colored red. As a result of this procedure, we observed that subgraphs exhibited within- and between-system functional interactions that were more likely than expected by chance.
(TIF)

S7 Fig Contrast of functional interactions between cognitive control tasks. We examine the relative differences in the distributions of mean strength of each pairwise functional interaction between task blocks of the Stroop task and task blocks of the Navon task. Using paired *t*-tests and FDR correction to account for multiple comparisons, we compare the strength of a functional interaction during the Stroop task to its strength during the Navon task, separately, for each positive correlation and for each negative correlation. To measure the difference in functional interaction strength, we compute the mean Fisher’s *r*-to-*Z* transformed correlation across subjects, separately for each positive correlation and for each negative correlation. Here, we plot the mean difference in Fisher’s *r*-to-*Z* value for functional interactions that exhibit a significant difference in their weight between the Stroop task and the Navon task as a symmetric adjacency matrix. A positive change in Fisher’s *r*-to-*Z* indicates a stronger effect of the functional interaction during the Stroop task than during the Navon task, and a negative change in Fisher’s *r*-to-*Z* indicates a stronger effect of the functional interaction during the Navon task than during the Stroop task.
(TIF)

S8 Fig Contrast of functional interactions between cognitive demand conditions. We examine the relative differences in the distributions of mean strength of each pairwise functional interaction between task blocks during the low cognitive demand condition and during the high cognitive demand condition, separately for the Stroop task and for the Navon task. Using paired *t*-tests and FDR correction to account for multiple comparisons, we compare the strength of a functional interaction during the low demand condition of a task to its strength during the high demand condition of the task, separately, for each positive correlation and for each negative correlation. To measure the difference in functional interaction strength, we compute the mean Fisher’s *r*-to-*Z* transformed correlation across subjects, separately for each positive correlation and for each negative correlation. Here, we plot the mean difference in Fisher’s *r*-to-*Z* value for functional interactions that exhibit a significant difference in their weight between the low cognitive demand condition and the high cognitive demand condition as a symmetric adjacency matrix. A positive change in Fisher’s *r*-to-*Z* indicates a stronger effect of the functional interaction during the high cognitive demand condition than during the low cognitive demand condition, and a negative change in Fisher’s *r*-to-*Z* indicates a stronger effect of the functional interaction during the low cognitive demand condition than during the high cognitive demand condition.
(TIF)
