# Correction: Subgraphs of functional brain networks identify dynamical constraints of cognitive control

**DOI:** 10.1371/journal.pcbi.1006420

**Published:** 2018-08-28

**Authors:** Ankit N. Khambhati, John D. Medaglia, Elisabeth A. Karuza, Sharon L. Thompson-Schill, Danielle S. Bassett

There is an error in the caption for Fig 4. *p* = 1.3^−7^ in the third sentence should read *p* = 1.3 x 10^−7^. Please see the complete, correct Fig 4 caption here.

**Fig 4:**
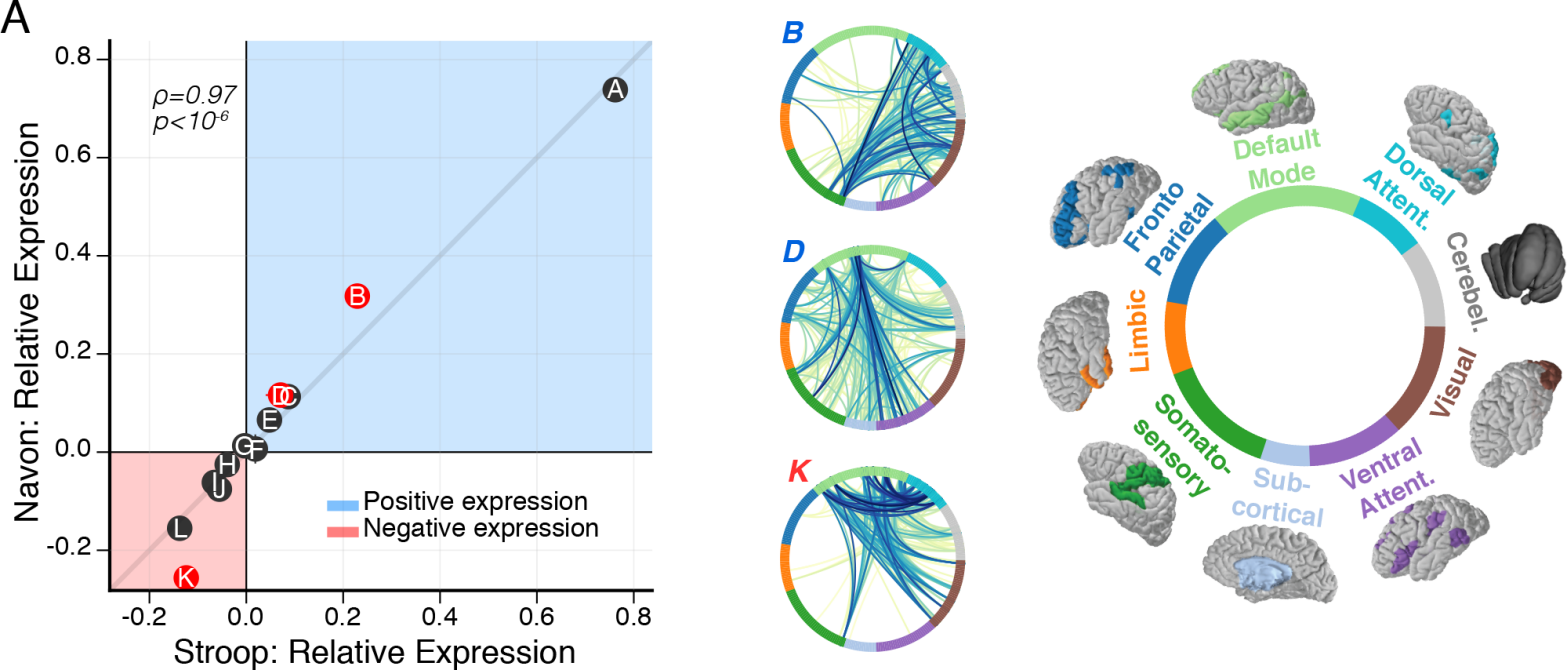
Subgraphs map functional interactions specific to cognitive control tasks. (*A*) Relationship between the relative subgraph expression during the Stroop task and the relative subgraph expression during the Navon task. Each point represents relative subgraph expression averaged over subjects, horizontal (vertical) error bars represent standard error of the mean for the Stroop (Navon) task. Generally, relative subgraph expression during the Stroop task is significantly associated with relative subgraph expression during the Navon task (Spearman's *ρ*, *ρ* = 0.97, *p* = 1.3 x 10^−7^), implying that subgraphs collectively follow similar rules of dynamical expression during the Stroop and Navon tasks. However, individual subgraphs may vary in the amount they are expressed during the Stroop and Navon tasks, which is signified by the perpendicular distance between a subgraph and the shaded gray line with slope equal to one. Using paired *t*-tests and FDR correction for multiple comparisons, we compare the distribution of relative subgraph expression between Stroop and Navon tasks across subjects. We find greater positive expression during the Navon task than the Stroop task for subgraph *B* (*t*_27_ = 4.4, *p* = 1.4 × 10^−4^) and subgraph *D* (*t*_27_ = 2.9, *p* = 7.0 × 10^−3^), and we find greater negative expression during the Navon task than the Stroop task for subgraph *K* (*t*_27_ = 5.1, *p* = 1.4 × 10^−5^). Thus, the rank of a subgraph in terms of its overall expression relative to other subgraphs is similar between the Stroop and Navon tasks, but its level of expression may be different depending on the task. Specifically, we find subgraphs *B* and *D* are more strongly associated with correlated dynamics during the Navon task than the Stroop task, and we find subgraph *K* is more strongly associated with anticorrelated dynamics during the Navon task than the Stroop task.

There is an error in the caption for Fig 5. *p* = 4.1^−9^ in the fifth sentence should read *p* = 4.1 x 10^−9^. Please see the complete, correct Fig 5 caption here.

**Fig 5:**
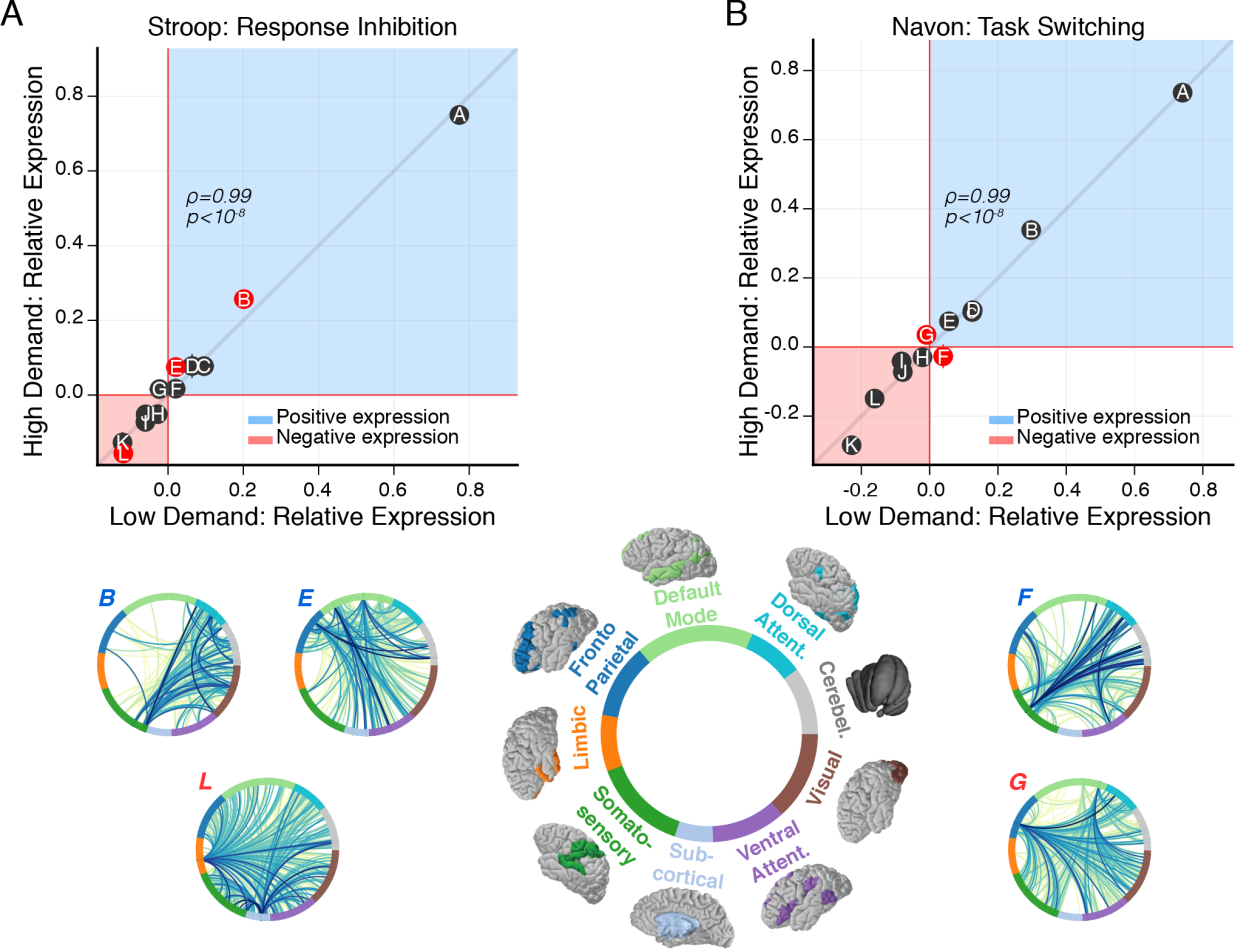
Modulation of subgraph expression coincides with increased cognitive demand. (*A*) Relationship between relative subgraph expression during the low cognitive demand condition and the high cognitive demand condition of the Stroop task. Each point represents relative subgraph expression averaged over subjects. Horizontal (vertical) error bars represent standard error of the mean for the low (high) demand condition. Similarly plotted for the low demand condition and high demand condition of the Navon task (shown in *B*). Generally, relative subgraph expression during the low demand condition is significantly associated with relative subgraph expression during the high demand condition for the Stroop task (Spearman's *ρ*, *ρ* = 0.99, *p* = 4.1 x 10 ^−9^) and for the Navon task (Spearman's *ρ*, *ρ* = 0.99, *p* = 4.1 × 10^−9^). These results imply that the rank of a subgraph in terms of its overall expression relative to other subgraphs is similar between the low cognitive demand condition and the high cognitive demand condition for the Stroop task and for the Navon task. To test whether individual subgraphs vary in the amount they are expressed as cognitive demand increases, we compare the distribution of relative subgraph expression between the low demand condition and the high demand condition for each task using paired t-tests and FDR correction for multiple comparisons. For the Stroop task, we find greater positive expression during the high demand condition than the low demand condition for subgraph *B* (*t*_27_ = 3.3, *p* = 2.7 × 10^−3^) and subgraph *E* (*t*_27_ = 3.2, *p* = 3.6 × 10^−3^), and we find greater negative expression during the high demand condition than the low demand condition for subgraph *L* (*t*_27_ = 2.5, *p* = 0.01). For the Navon task, we find greater positive expression during the high demand condition than the low demand condition for subgraph *G* (*t*_27_ = 2.9, *p* = 8.2 × 10^−3^), and we find greater negative expression during the high demand condition than the low demand condition for subgraph *F* (*t*_27_ = 2.7, *p* = 0.01). These results collectively suggest that subgraph expression shifts alongside changes in cognitive demand in a manner that is specific to each cognitive task. Specifically, the change in subgraph expression that accompanies an increase in cognitive demand may involve an increase in correlated or anticorrelated dynamics. These dynamics potentially implicate an antagonistic network mechanism of cognitive demand whereby one set of subgraphs engage through more positive expression while another set of subgraphs disengage through more negative expression.

There is missing information in reference 40. The correct reference is: Olshausen BA, Field DJ. Emergence of simple-cell receptive field properties by learning a sparse code for natural images. Nature. 1996; 381: 607–609.

There is missing information in reference 54. The correct reference is: Bassett DS, Khambhati AN. A network engineering perspective on probing and perturbing cognition with neurofeedback. Ann N Y Acad Sci. 2017; 1396(1): 126–143.
