# Supplementary figures and images for: Subgraphs of functional brain networks identify dynamical constraints of cognitive control
Source: PLoS Comput Biol. 2018 Jul 6;14(7):e1006234. doi: 10.1371/journal.pcbi.1006234 (PMC6056061; doi:10.1371/journal.pcbi.1006234)

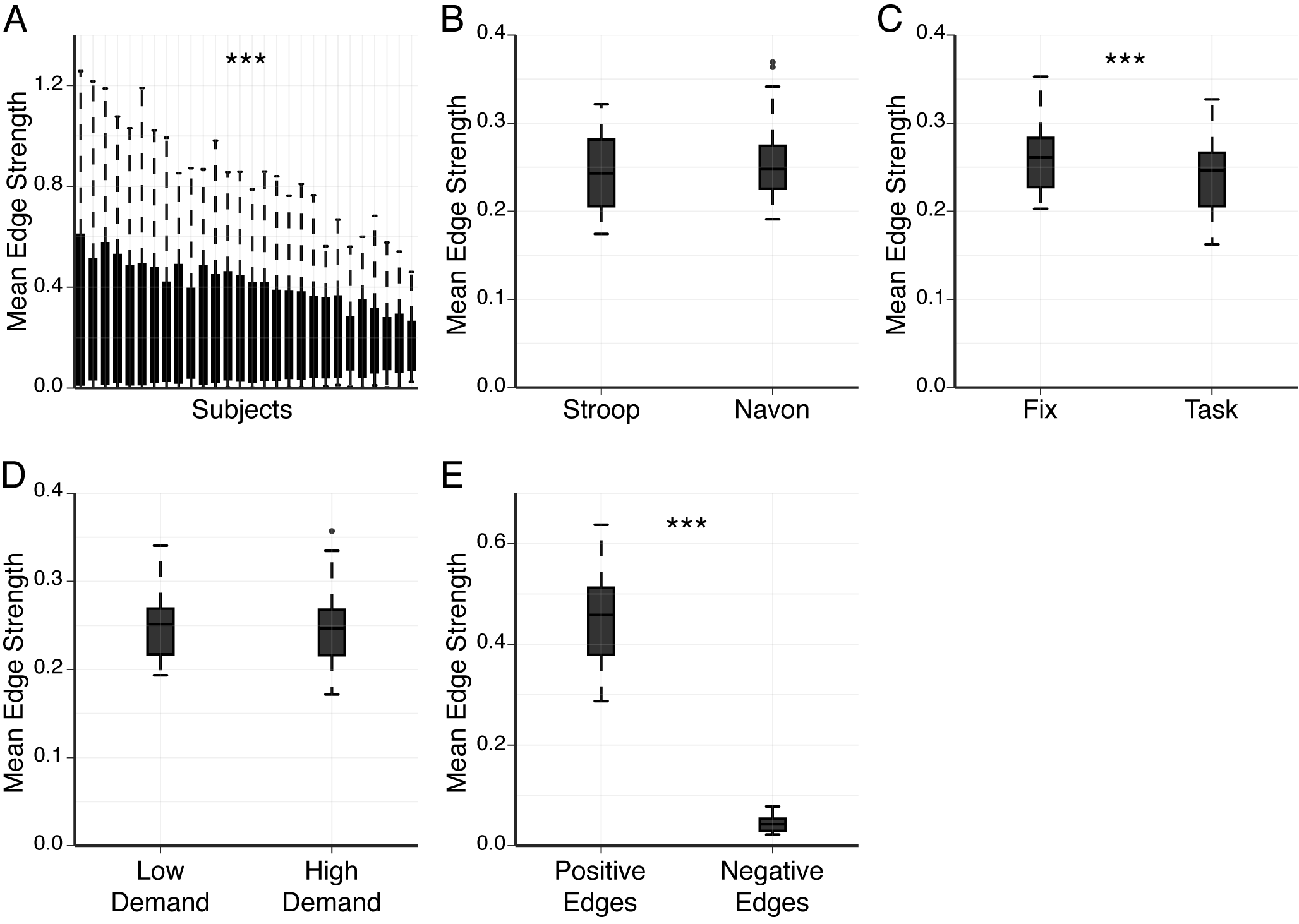

Supplement: S1 Fig — (A) Distribution of edge weights across all experimental blocks for each of the 28 participants in the study. We find a significant difference in the edge weights between subjects (one-way ANOVA; F = 2.5, p = 3.5 × 10^−5^). (B) Distribution of mean edge weight across subjects for each cognitive control task. We find no significant difference in mean edge strength across subjects between blocks during the Stroop task and blocks during the Navon task (paired t-test, t27 = −1.5, p = 0.14). (C) Distribution of mean edge weight across subjects for fixation blocks and task blocks. We find a significant decrease in mean edge strength across subjects between blocks during the fixation period and blocks during the cognitive control task period (paired t-test, t27 = 4.7, p6.3 × 10−5). (D) Distribution of mean edge weight across subjects for low demand blocks and for high demand blocks. We find no significant difference in mean edge strength across subjects between blocks during the low cognitive demand conditions and blocks during the high cognitive demand conditions (paired t-test, t27 = 0.35, p = 0.73). (E) Distribution of mean magnitude edge weight for positive correlations and negative correlations across subjects. We find that the magnitude of negative edge weights is significantly lower than the magnitude of positive edge weights (paired t-test; t27 = 20.0, p = 9.7 × 10^−18^). (TIF) [file pcbi.1006234.s001.tif]

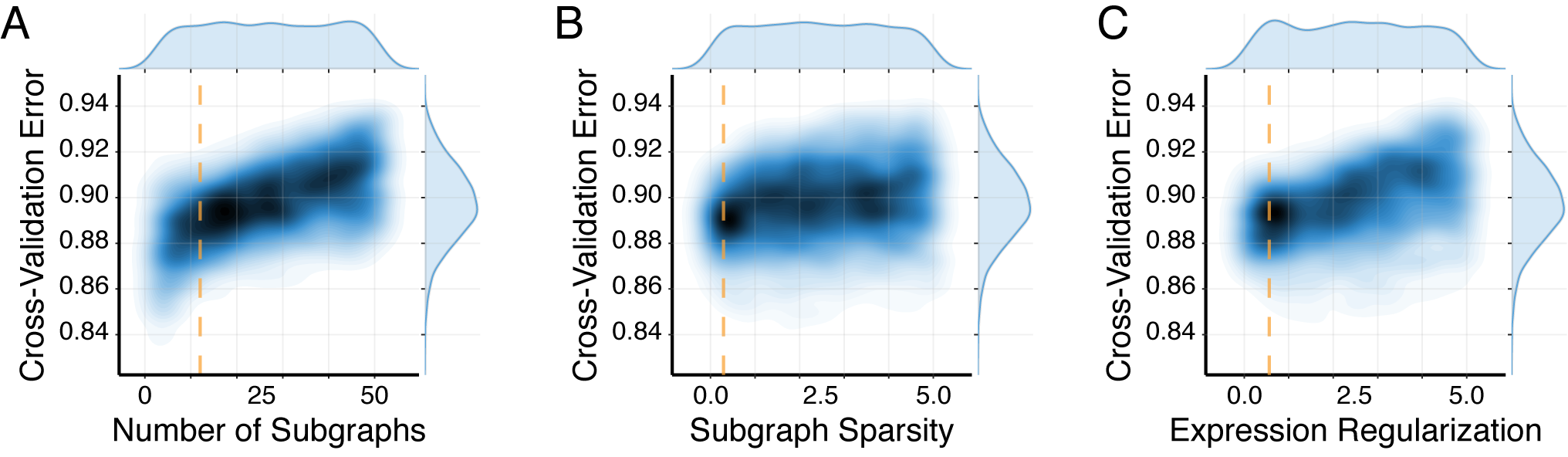

Supplement: S2 Fig — (A-C) NMF-based subgraph detection requires optimizing three parameters: the number of subgraphs m, the sparsity of subgraph edge weights β, and the regularization of temporal expression coefficients α. To characterize this parameter space, we randomly sampled m, β, and α from a three-dimensional uniform distribution (m ∈ [3, 50], β ∈ [0.01, 5.0], α ∈ [0.01, 5.0]) and applied NMF to the configuration matrix using each parameter set. Kernel density estimate of each bivariate distribution is indicated by the contour plot, where darker shades of blue indicate greater probability mass of the random sampling distribution. Optimal parameters are the average parameter values that yielded cross-validation error in the bottom 5% of the sampling distribution and are indicated by the dashed orange line. (TIF) [file pcbi.1006234.s002.tif]

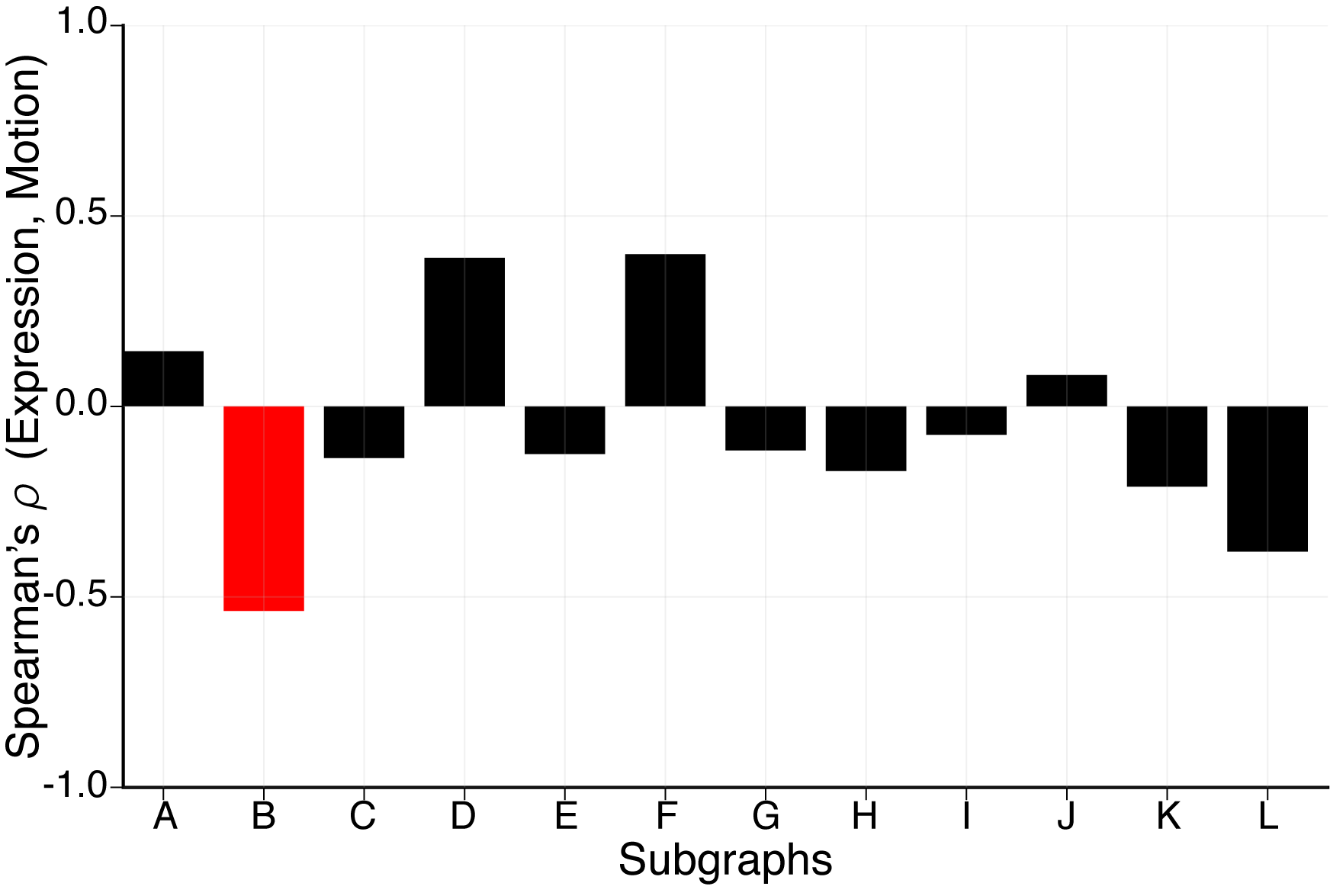

Supplement: S3 Fig — We test whether a functional subgraph is confounded by motion artifact by estimating the correlation between the mean subgraph expression and the mean motion score over subjects. Using a Spearman’s ρ and FDR correction for multiple comparisons, we find that the expression of subgraph B decreases with increasing motion (ρ = −0.53, p = 3.2 × 10^−3^). (TIF) [file pcbi.1006234.s003.tif]

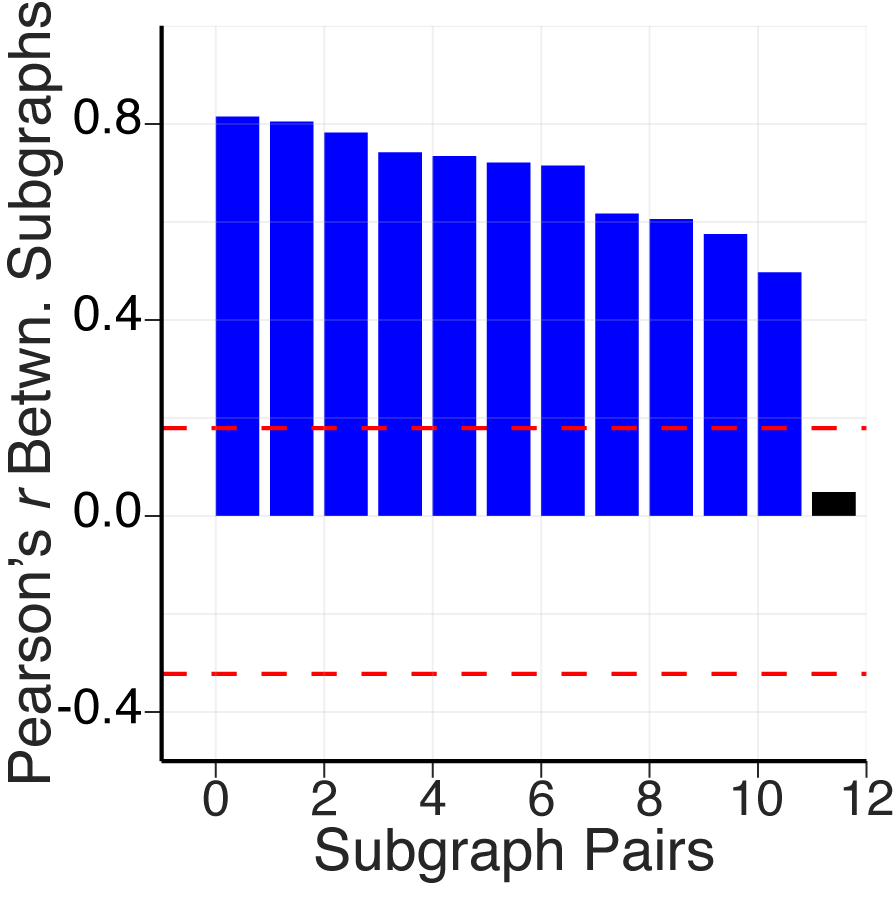

Supplement: S4 Fig — Test-retest reliability of subgraphs decomposed from the first three experimental blocks and second three experimental blocks of the cognitive control dataset. NMF was separately applied to each split dataset to identify two sets of subgraphs. Subgraphs were uniquely paired between the two datasets using the Hungarian assignment algorithm [74] and the minimum Euclidean distance between subgraph edge vectors as a measure of assignment cost. To quantify subgraph reliability, we computed the Pearson correlation between pairs of assigned subgraphs and ranked subgraph pairs in decreasing order of correlation. We generated a null distribution of correlations between all possible non-assigned subgraph pairs (indicated by red dashed lines at the 95% confidence interval). Subgraphs with significantly greater correlation than expected by the null distribution are colored blue (p < 0.05; Bonferroni corrected for multiple comparisons). We found eleven of twelve subgraph pairs were more reliable than expected by chance assignment across the split dataset. (TIF) [file pcbi.1006234.s004.tif]

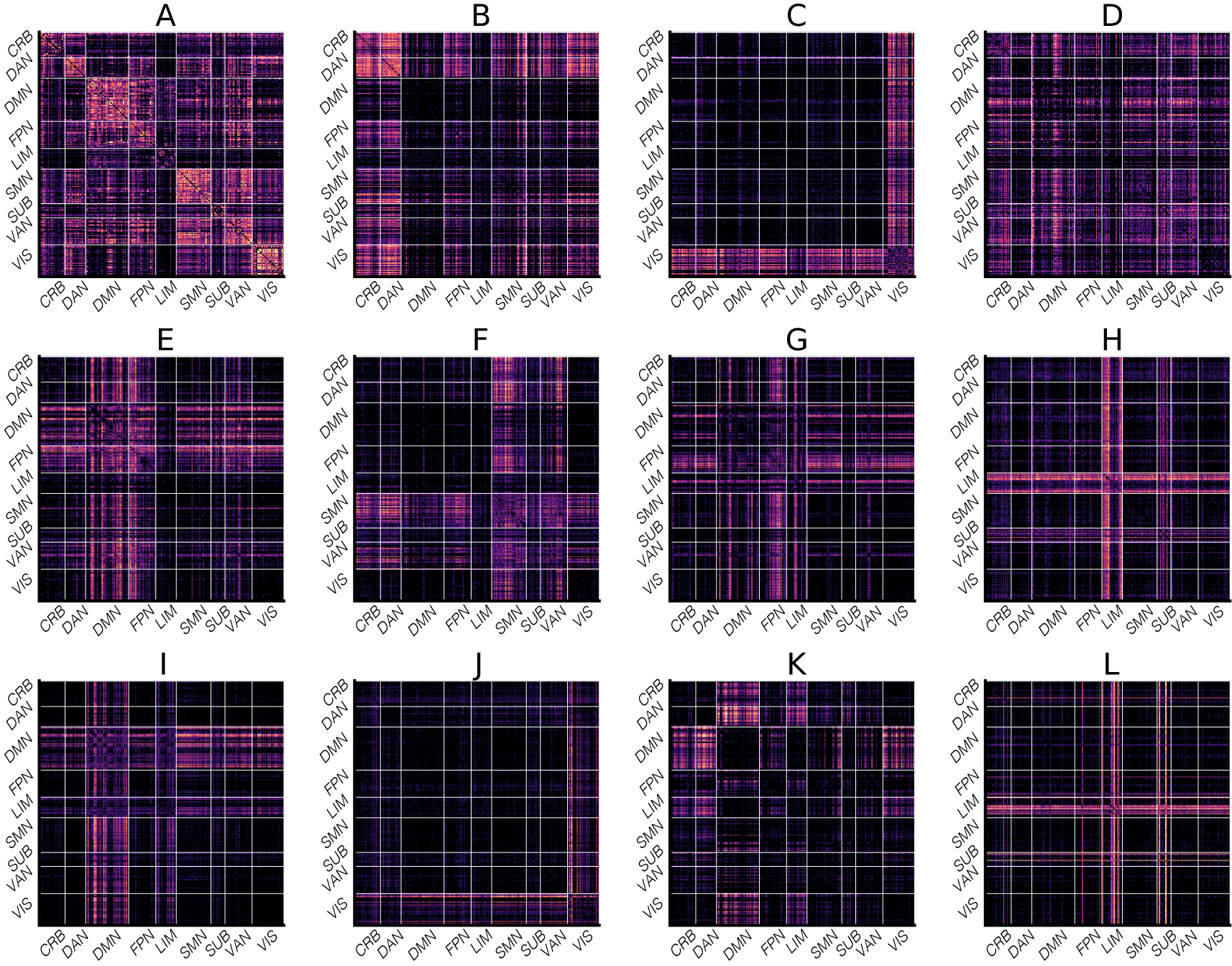

Supplement: S5 Fig — We visualized the edge weights associated with a functional subgraph as a symmetric and fully-weighted adjacency matrix with size 262 × 262, where 262 is the number of nodes in the functional network. Based on the assignment of each of the 262 brain regions into one of nine putative cognitive systems [33]—dorsal attention (DAN), default mode (DMN), frontoparietal (FPN), limbic (LIM), somatosensory (SMN), subcortical (SUB), ventral attention (VAN), visual (VIS), and cerebellum (CRB)—we reorganize the rows and columns of each adjacency matrix such that nodes assigned to the same cognitive system are contiguously ordered. We observe subgraphs whose strongest edges (lighter shades of purple) tend to fall within well-defined boundaries of known cognitive systems. (TIF) [file pcbi.1006234.s005.tif]

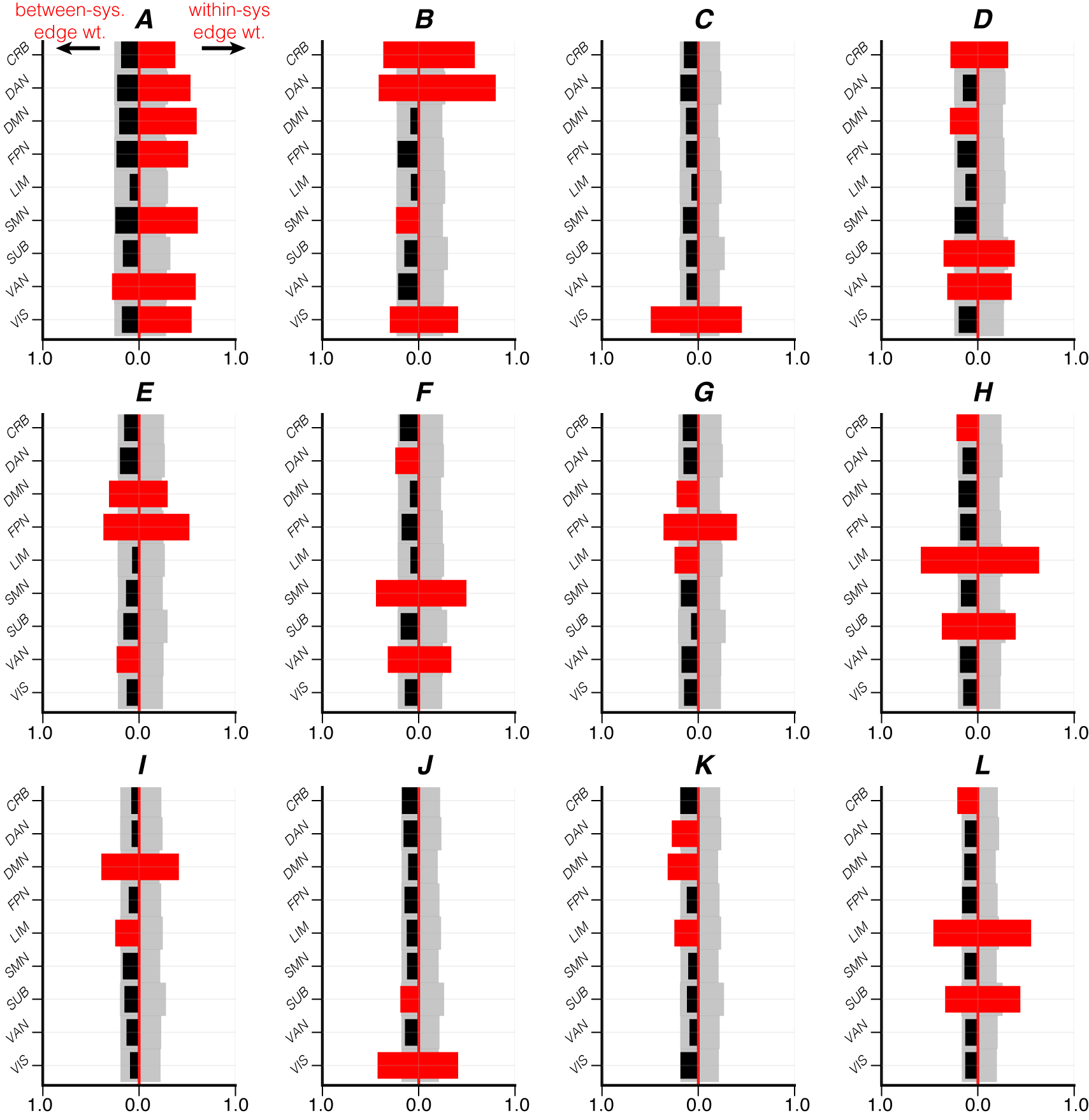

Supplement: S6 Fig — We determined whether functional subgraphs reflect functional interactions within and between known cognitive brain systems using a previously documented approach [22]. Based on the assignment of each of the 262 brain regions into one of nine putative cognitive systems [33]—dorsal attention (DAN), default mode (DMN), frontoparietal (FPN), limbic (LIM), somatosensory (SMN), subcortical (SUB), ventral attention (VAN), visual (VIS), and cerebellum (CRB)—we computed the mean subgraph edge weight between brain regions within the same cognitive system (within-system edge weight) and mean subgraph edge weight between brain regions of one system to brain regions in all other systems (between-system edge weight). Here, we plot the mean within-system edge weight and the mean between-system edge weight for each cognitive system and each functional subgraph as horizontal bars; the mid-line implies a mean edge weight of zero, bars to the left of the midline correspond to mean between-system edge weight, and bars to the right of the midline correspond to mean within-system edge weight. Error bars correspond to standard error of the mean edge weight. To assess whether a within-system or between-system edge weight was more likely observed due to the topology of the subgraph than expected by chance, we generated a null distribution for each system-level interaction for each subgraph by permuting a subgraph’s edge weights between nodes 10000 times and recomputing the average edge weight for each permutation. We then compared each true mean edge weight to the null distribution (shaded in gray) and retained only significant within-system and between-system edge weights (p < 0.05; Bonferroni corrected for multiple comparisons). Cognitive systems with significant within-system edge weight or significant between-system edge weight are colored red. As a result of this procedure, we observed that subgraphs exhibited within- and between-system functional interactions that were more likely th [file pcbi.1006234.s006.tif]

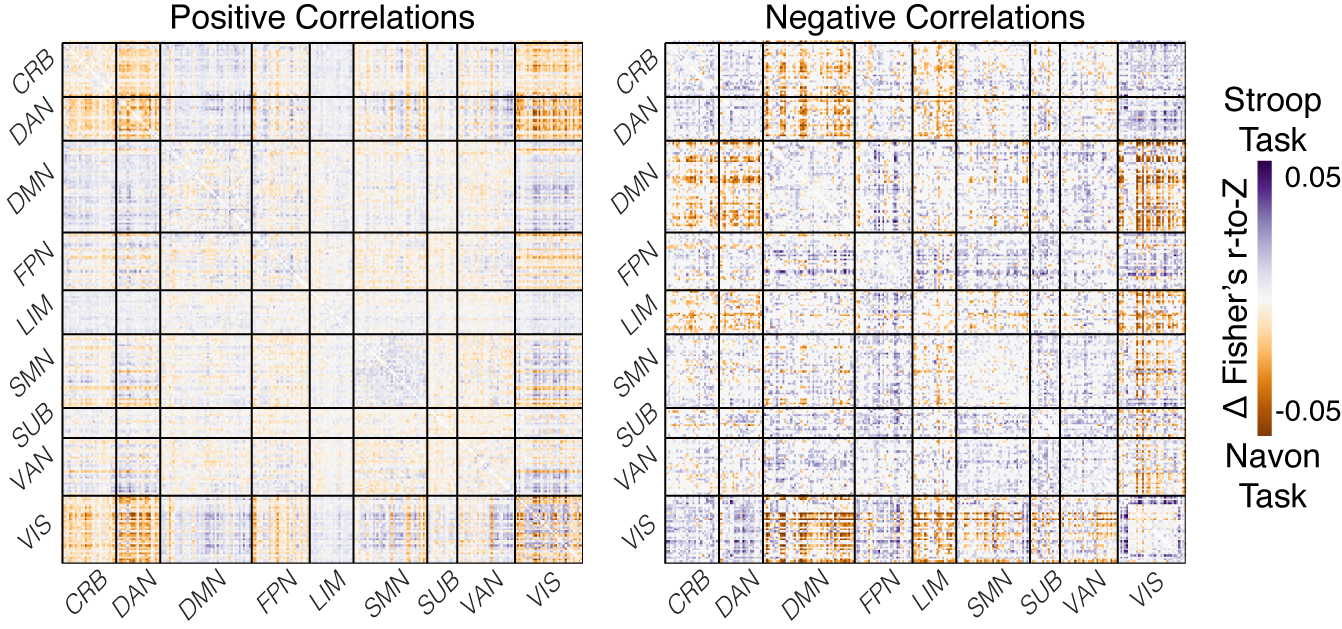

Supplement: S7 Fig — We examine the relative differences in the distributions of mean strength of each pairwise functional interaction between task blocks of the Stroop task and task blocks of the Navon task. Using paired t-tests and FDR correction to account for multiple comparisons, we compare the strength of a functional interaction during the Stroop task to its strength during the Navon task, separately, for each positive correlation and for each negative correlation. To measure the difference in functional interaction strength, we compute the mean Fisher’s r-to-Z transformed correlation across subjects, separately for each positive correlation and for each negative correlation. Here, we plot the mean difference in Fisher’s r-to-Z value for functional interactions that exhibit a significant difference in their weight between the Stroop task and the Navon task as a symmetric adjacency matrix. A positive change in Fisher’s r-to-Z indicates a stronger effect of the functional interaction during the Stroop task than during the Navon task, and a negative change in Fisher’s r-to-Z indicates a stronger effect of the functional interaction during the Navon task than during the Stroop task. (TIF) [file pcbi.1006234.s007.tif]

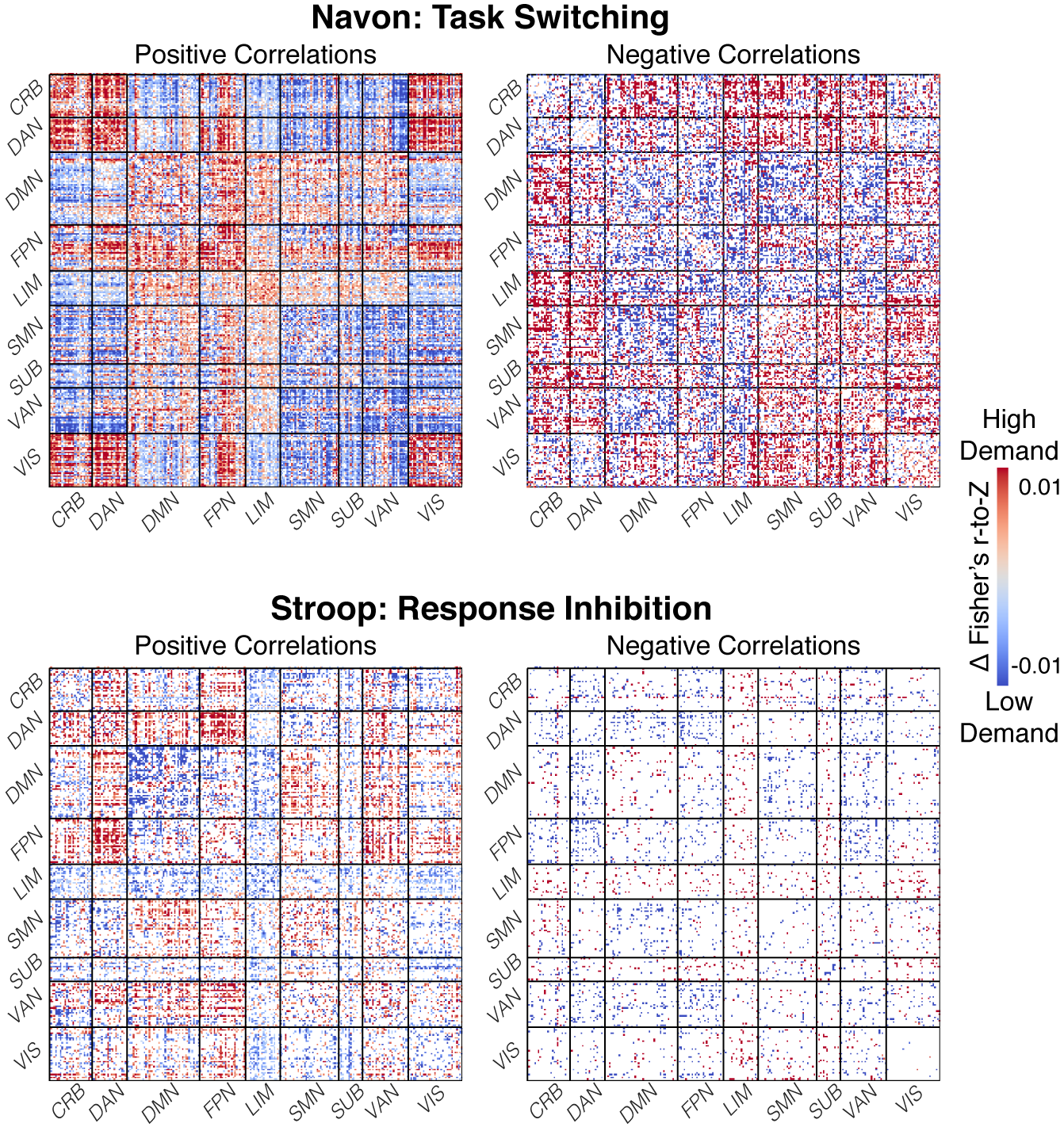

Supplement: S8 Fig — We examine the relative differences in the distributions of mean strength of each pairwise functional interaction between task blocks during the low cognitive demand condition and during the high cognitive demand condition, separately for the Stroop task and for the Navon task. Using paired t-tests and FDR correction to account for multiple comparisons, we compare the strength of a functional interaction during the low demand condition of a task to its strength during the high demand condition of the task, separately, for each positive correlation and for each negative correlation. To measure the difference in functional interaction strength, we compute the mean Fisher’s r-to-Z transformed correlation across subjects, separately for each positive correlation and for each negative correlation. Here, we plot the mean difference in Fisher’s r-to-Z value for functional interactions that exhibit a significant difference in their weight between the low cognitive demand condition and the high cognitive demand condition as a symmetric adjacency matrix. A positive change in Fisher’s r-to-Z indicates a stronger effect of the functional interaction during the high cognitive demand condition than during the low cognitive demand condition, and a negative change in Fisher’s r-to-Z indicates a stronger effect of the functional interaction during the low cognitive demand condition than during the high cognitive demand condition. (TIF) [file pcbi.1006234.s008.tif]
